# Development of the national consensus statement on ear health and hearing check recommendations for Aboriginal and Torres Strait Islander children aged under 6 years attending primary care: systematic scoping review and e-Delphi

**DOI:** 10.1186/s12875-024-02307-6

**Published:** 2024-03-14

**Authors:** Samantha Harkus, Vivienne Marnane, Isabel O’Keeffe, Carmen Kung, Meagan Ward, Neil Orr, John Skinner, Jessica Kate Hughes, Lose Fonua (Wiradjuri), Michelle Kennedy (Wiradjuri), Kelvin Kong (Worimi), Mary Belfrage

**Affiliations:** 1 National Acoustic Laboratories, Sydney, Australia; 2 Macquarie University, Sydney, Australia; 3 The University of Sydney Library, Sydney, Australia; 4 The University of Melbourne, Melbourne, Australia; 5 The University of Newcastle, Newcastle, Australia; 6 Department of Medicine, Royal Melbourne Hospital, University of Melbourne, Melbourne, Australia

**Keywords:** Consensus, Recommendations, Ear health checks, Hearing, Early identification, Aboriginal and Torres Strait Islander, Child health, Primary health, Otitis media, Systematic scoping review

## Abstract

**Background:**

Early detection of long-term, often asymptomatic, middle ear infection in young Aboriginal and Torres Strait Islander children is more likely to be achieved when ear health and hearing checks are routinely undertaken in primary healthcare. Evidence consistently demonstrates the adverse impacts of this condition on the development and wellbeing of children and their families. We aimed to develop feasible, evidence- and consensus-based primary healthcare recommendations addressing the components and timing of ear health and hearing checks for Aboriginal and Torres Strait Islander children aged under 6 years, not already known to have, nor being actively managed for, ear and hearing problems.

**Methods:**

A 22-person working group comprising Aboriginal and Torres Strait Islander and non-Indigenous members from the primary healthcare, ear, hearing, and research sectors provided guidance of the project. A systematic scoping review addressed research questions relating to primary health ear health and hearing checks for Aboriginal and Torres Strait Islander and other populations at increased risk of persistent ear health problems. Twelve primary studies and eleven guidelines published between 1998 and 2020 were identified and reviewed. Quality and certainty of evidence and risk of bias ratings were completed for studies and guidelines. In the absence of certain and direct evidence, findings and draft recommendations were presented for consensus input to a 79-member expert panel using a modified e-Delphi process. Recommendations were finalised in consultation with working group members and presented to expert panel members for input on considerations relating to implementation.

**Results:**

Overall, the quality, certainty, and directness of evidence in the studies and guidelines reviewed was low. However, the findings provided a basis and structure for the draft recommendations presented during the consensus-building process. After two e-Delphi rounds, seven goals and eight recommendations on the components and timing of Ear Health and Hearing Checks in primary healthcare for young Aboriginal and Torres Strait Islander children were developed.

**Conclusions:**

The systematic scoping review and consensus-building process provided a pragmatic approach for producing strong recommendations within a reasonably short timeframe, despite the low quality and certainty of evidence, and paucity of studies pertaining to primary healthcare settings.

**Supplementary Information:**

The online version contains supplementary material available at 10.1186/s12875-024-02307-6.

## Background

Otitis media (OM) or middle ear infection/inflammation continues to be prevalent among young Aboriginal and Torres Strait Islander children. Up to one in two children aged younger than 3 years in urban areas, and up to nine in ten in remote areas, experience the condition [1–4]. Children whose first episode commences prior to 6 months of age are more likely to develop persistent forms of OM [1, 4]. Relative to non-Indigenous Australian children, presentations of OM in Aboriginal and Torres Strait Islander children are typically more severe and persistent, start earlier and last longer, and have greater impact on hearing [5, 6]. The factors driving these prevalence rates relate strongly to social determinants and environmental factors that are a legacy of colonisation, racism, and disempowering government policies, such as inadequate housing, economic disadvantage, and poor access to healthcare services [7].

For affected children, early and persistent OM interferes with communication experiences, and thus with development of listening and communication skills, foundational for literacy [8–10]. Three Australian data-linkage studies found a link between OM-related hearing loss in early childhood and delay across multiple developmental domains at school entry [11–13]. Furthermore, caregiver and family wellbeing is affected by ongoing concern for the child, and frustration at both delays in recognition of persistent OM and in receiving effective healthcare [14, 15].

To avert or reduce these impacts, early identification of persistent forms of OM is critical. As the most common form of OM (OM with effusion, OME) is often asymptomatic, routine and proactive ear health checks are essential and recommended [16, 17].

In practice, however, ear health checks for Aboriginal and Torres Strait Islander children are undertaken neither proactively nor routinely [14, 18]. Checks are often initiated by parent or caregiver concern or request, and published health protocols and resources reveal substantial variation in the recommended components and timing of ear health and hearing checks for Australian children, including for Aboriginal and Torres Strait Islander children [19–22].

This study addressed the need for consistency across Australian primary health systems on the components and timing of ear health and hearing checks for Aboriginal and Torres Strait Islander children during early childhood. Early and regular checks are essential so that early diagnosis, active treatment, and tracking can be initiated and children and their families can be supported with actions that mitigate the negative effects of OM.

The study aimed to develop national ear health and hearing check (EHHC) recommendations for Australian primary healthcare practitioners, to guide effective assessment of ear health and hearing status of Aboriginal and Torres Strait Islander children aged under 6 years attending primary care, who are not known to have, or are not being actively managed for, ear health and hearing problems. This paper focuses on the process undertaken to develop the recommendations. A companion paper that describes the recommendations in detail as well as key practice changes required for implementation is also available [23].

## Methods

### Study design and setting

**Fig. 1:**
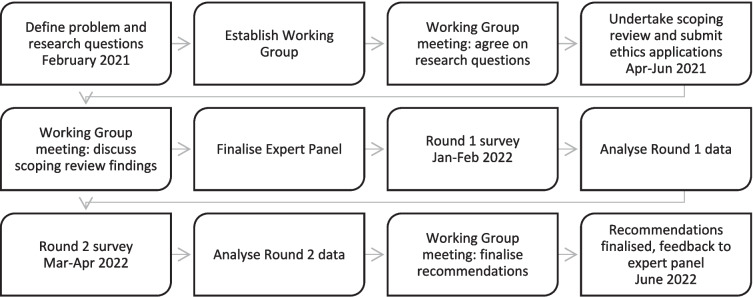
Overview of Ear Health and Hearing Check project process

The project commenced in February 2021 and ended in June 2022. It involved a systematic scoping review of Australian and international literature relevant to the components and timing of ear health and hearing checks in primary healthcare settings, followed by a modified e-Delphi consensus process [24], undertaken with a large, national expert panel. The use of an e-Delphi (electronic Delphi) was well suited to the panel size and nature, providing members with a more flexible timeframe for participation across several time zones. Figure 1 provides an overview of the process.

### Project team and working group

The project team comprised Aboriginal and non-Indigenous researchers experienced in the provision of clinical care and/or health-related research with Aboriginal and Torres Strait Islander communities, and included a general practitioner, public health researchers, and clinician-researchers from the ear health and hearing sector. A research librarian assisted in the design of the literature search strategy.

A 22-member working group was established for the project. Invitees to the group were people with cultural, clinical, and/or research expertise in primary healthcare or in Aboriginal and Torres Strait Islander children’s ear and hearing health. Members were Aboriginal and/or Torres Strait Islander (46%) and non-Indigenous, and over half (59%) worked in primary health settings. Four meetings were held with the working group during the project, during which members provided guidance on research questions for the scoping review, establishment of the expert panel, interpretation of research findings, resolution of lack of consensus, and formulation of the final recommendations. Meetings were held online. All 22 members attended at least one meeting and an average of 13 members attended each meeting. Six members attended 3 or 4 meetings.

### Systematic scoping review research questions and scope

The systematic scoping review addressed the research questions shown in Table 1. The protocol was registered on the Open Science Framework (https://osf.io/259ae/), an open-source web platform that facilitates open collaboration in science research [25].

**Table 1:** Systematic scoping review research questions

**Systematic scoping review research questions**
For children aged under 6 years:
1. What is the evidence on efficacy of tools to screen middle ear status, including sensitivity and specificity?
2. What is the evidence on efficacy of tools to screen hearing and listening, including sensitivity and specificity?
3. What are the recommended components for ear and hearing checks?
4. What are the key ages for ear and hearing checks?

Evidence relating to the total Australian population, and to Aboriginal and Torres Strait Islander peoples specifically, was in-scope, as well as evidence from international populations, particularly First Nations, that may be applicable to Aboriginal and Torres Strait Islander peoples. Evidence relating to newborn hearing screening and school-based hearing screening was outside the scope of the review.

Published studies, including meta-analyses, systematic reviews, primary studies, relevant guidelines, and health sector resources were reviewed for evidence on ear health and hearing assessment activities in primary healthcare, for information on effectiveness of these activities where available, and on the timing of the activities. The quality and certainty of the evidence was assessed using the Grading of Recommendations, Assessment, Development and Evaluations (GRADE) framework for primary studies [26] and the AGREE-II for guidelines [27]. Risk of bias was assessed using QUADAS-2 for primary studies [28] and ROBIS for the systematic review [29].

### Search strategy

The scoping review search strategy focused on research questions 1 and 2 and was developed in collaboration with a librarian. The search was structured using the concepts ‘children under 6 years of age’ AND ‘ear and hearing screening tools’ AND ‘primary health care settings’. A comprehensive number of electronic databases was searched: Medline (Ovid), Embase (Ovid), Cochrane Database of Systematic Reviews (Ovid), Cochrane Central Register of Controlled Trials (Ovid), CINAHL (EbscoHost), Scopus, and nine health related indexing databases on Informit. The electronic database searches were performed by the librarian on 14-16 July 2021. A publication date limit of 2000-current and journal article limit were applied in Informit databases only. Search terms are available as Supplementary information. Websites targeted for grey literature included Australian state and territory health departments, Australian Indigenous HealthInfoNet [30], Lowitja Institute [31], and Clinical Guidelines [32] .

Extracted data included: year, authors, publication title, research question or study purpose, study design, settings, age of participants, sample size, screening test (sensitivity, specificity), level of evidence and quality, and feasibility requirements. Two authors extracted data from all included studies into an Excel spreadsheet, with other authors double-checking a sample of the extraction. Any disagreements were resolved through discussion with a third reviewer. We used the Covidence systematic review management software to manage the data arising from our searches [33].

In addition, relevant guidelines, policy documents, and parent/caregiver resources published by Australian state and territory health departments assisted with answering questions 3 and 4. These documents were either accessed by web search or shared directly with the research team upon request.

### Expert panel

A large, national expert panel was established for the consensus-building (e-Delphi) phase of the project. Recruitment was strategic, and occurred as follows:

1. All working group members were invited.
2. Project team and working group members were invited to identify known Aboriginal, Torres Strait Islander, and non-Indigenous practitioners working with Aboriginal and Torres Strait Islander children and families in community-controlled or mainstream healthcare with expertise in ear health and hearing, including working in primary health, audiology, otolaryngology, paediatrics, and speech pathology, and as researchers.
3. Written invitations to nominate a representative for the e-Delphi process were sent to state/territory health services and to peak bodies representing Aboriginal and Torres Strait Islander health, Aboriginal and Torres Strait Islander doctors, health workers and health practitioners, and to national colleges representing general practitioners and surgeons. Organisations were provided with criteria to assist with identifying an appropriate invitee.

Recruitment ceased once there was approximately proportional participation from all Australian geographical and remoteness areas, from community controlled and mainstream primary healthcare sectors, and of Aboriginal and/or Torres Strait Islander experts from as many sectors, professions, jurisdictions, and remoteness areas as possible. In addition to the 22 working group members, 57 (of 69) invitees accepted the invitation to join the expert panel.

### Survey development and administration

Two rounds of a modified e-Delphi survey were developed by the project team. The first survey included background and rationale for the development of recommendations, clarification on intended users and target patient population, and on the scope of the recommendations. Seven goals of routine Ear Health and Hearing Checks, informed by the evidence review, were presented to the expert panel. Participants were invited to indicate on a 4-point Likert scale [34] the extent to which they agreed with each goal, or select ‘I don’t feel qualified to answer’ or ‘I need more information to be able to decide’. The panel was also invited to comment on the goals and to propose additional ones. Further, eight draft Ear Health and Hearing Check recommendations were presented. Each was accompanied by a summary of the evidence, grading of evidence quality, as well as a rationale for the recommendation. Survey participants were asked to indicate, also on a 4-point Likert scale, the extent to which they agreed or disagreed with each recommendation or select ‘I don’t feel qualified to answer’ or ‘I need more information to be able to decide’, and invited to comment on the draft recommendations. Panel members were also asked to rate, on a 4-point Likert scale, how feasible they felt the recommendation would be to implement in a primary healthcare setting. The goals and recommendations were reviewed and revised following this feedback and presented again to the panel in Round 2, with a summary of Round 1 findings and rationales for revisions.

Study data were collected and managed using REDCap (Research Electronic Data Capture) electronic data capture tools hosted at the National Acoustic Laboratories [35, 36]. REDCap is a secure, web-based software platform designed to support data capture for research studies. At the commencement of the consensus-building process, all (79) expert panel members received an email with an embedded REDcap link that allowed access to the survey, participant information, and the summary of evidence. Panel members were asked to keep the link confidential. Information on how the privacy of participants would be protected and how to raise concerns was set out in the email text. They were also given an estimate of completion time (up to 60 minutes), and instructions for completing the survey in stages. They were given six weeks to complete the survey. Members were reminded that participation was anonymous and voluntary, that selecting ‘next’ at the end of the introduction page of the survey would indicate their informed consent, and that they could withdraw at any time by closing the survey. Periodic email reminders were sent during the response period, which included progress updates on number of surveys completed. Following survey closure, level of consensus was calculated based on ‘Agree’ and ‘Strongly agree’ responses. Comments were collated and analysed thematically by the research team using an inductive approach, adhering to processes outlined by Braun and Clarke [37, 38]. The themes generated were presented to the expert panel in the following round of the e-Delphi.

Round 2 of the e-Delphi was administered in the same way. All expert panel members were invited to take part in the second round. Throughout the second survey, links enabled access to the supporting information provided for Round 1 and to the summary of findings from Round 1, both also available as pre-reading. Rationales accompanied each revised recommendation.

### Finalising recommendations, resolving lack of consensus, and providing feedback

By the end of Round 2 of the e-Delphi, consensus was reached for all goals and all but one recommendation which related to the inclusion of audiometry in the checks. The outstanding recommendation was resolved after two meetings, and a follow-up email discussion, with working group members. Subsequently, all expert panel members were invited to one of two feedback meetings, at which the final recommendations were presented. At these meetings, panel members were invited to consider and discuss barriers and enablers to implementation into practice in primary healthcare. Twenty-three members attended the first feedback meeting, and 14 members attended the second; 32 members attended at least one meeting. All panel members received a copy of the information presented at these meetings, as well as a collated summary of the barriers and enablers put forward by panel members during the two meetings.

### Ethical considerations

All methods were carried out in accordance with relevant ethical guidelines and regulations [39, 40]. Expert panel members were provided with information on context and aims of the study, how their privacy would be protected, how to raise concerns and contact the project team for further information. Members were informed how to indicate consent and of their rights in relation to participation and withdrawal.

In relation to ethical considerations when undertaking research relating to Aboriginal and Torres Strait Islander people and communities, we demonstrated spirit and integrity through ensuring that Aboriginal and Torres Strait Islander technical, clinical, and cultural knowledge were embedded throughout the project. We demonstrated credibility of intent through respectful engagement with Aboriginal and Torres Strait Islander people and organisations and ensuring equity of voice in project design and implementation. Team members recognised Aboriginal and Torres Strait Islander understandings of social and emotional wellbeing, the interconnectedness of health, place, and culture, the destructive impacts of colonisation on individual and collective social, emotional, and physical wellbeing, and the strengths of communities, families, and individuals. The project was informed by the National Health and Medical Research Council’s guidelines on ethical conduct in research with Aboriginal and Torres Strait Islander peoples and communities [40], the NSW Aboriginal Health and Medical Research Council’s health ethics key principles [39], and the CREATE checklist for assessing the quality of health research from an Indigenous perspective [41]. All participants in the e-Delphi consensus process provided informed consent.

Ethics approvals for the e-Delphi study were received from the Aboriginal Health and Medical Research Council (NSW) (1858/21), the Western Australian Aboriginal Health Ethics Committee (HREC1108), the Aboriginal Health Research Ethics Committee (SA) (04-21-944), the Menzies School of Health Research (NT) (HREC 2021-4137), and the Hearing Australia Human Research Ethics Committee (HAHREC 2021-07).

## Results

### Findings of systematic scoping review

**Fig. 2:**
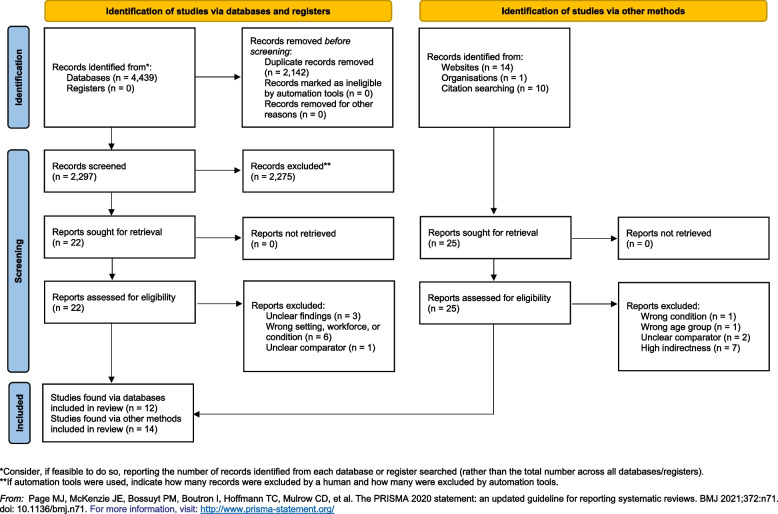
Flowchart for articles included in the evidence review

In total, 26 studies were included in the review. A systematic database search (undertaken by JH, JS, and NO) identified 22 studies published between 2000 to 2021, ten of which were subsequently excluded because of an incorrect target condition, setting, workforce, or lack of clarity in the presentation of findings. Furthermore, researchers (SH, VM, CK, MW, NO, IO) identified 25 studies via other methods, 11 of which were excluded for reasons relating to target condition or workforce, comparator, or level of directness (Fig. 2).

The 12 remaining studies compared tests against an established standard (usually standard otoscopy and audiometry). Most studies were non-randomised comparative studies. One study was a randomised controlled trial [42], and one paper was a systematic review [43] (see Table 2). Two studies concerning ear health and hearing screening related to Aboriginal and Torres Strait Islander populations [44], and seven related to other Indigenous populations or to low resource settings, including South Africa [45], Kenya [46], and India [47]. Heterogeneity among the studies related to study populations, study designs, and healthcare settings. Table 2 provides an overview of the included papers.

**Table 2:** Comparison of tools for screening ear health and hearing

**Tools for screening ear health**					
**Author/year and *study type***	**Sample**	**Study setting**	**Ref test**	**Comparator test**	**Key findings and effect measures**
Chianese et al, 2007 [48] *Non-randomised comparative study*	786 healthy children aged 2-24 months	Primary care, US	Tympanometry and Spectral Grade Acoustic Reflectometry (SGAR)	Pneumatic otoscopy	- Tympanometry slightly more discerning than SGAR in predicting middle ear fluid: tympanometry AUC = 0.83 and SGAR AUC = 0.78 - 95%CI not reported
Helenius et al, 2012 [49] *Non-randomised comparative study*	515 children 0.5-3 years (mean 16 months)	Primary health care, Finland	Tympanometry	Pneumatic otoscopy	- All peaked tympanograms could be taken as healthy middle ears in primary care - Tympanometry unable to differentiate acute OM (AOM) from OM with effusion (OME) - For asymptomatic children: when middle ear aerated, tympanogram peaked in 97% of exams; when AOM diagnosed, tympanogram flat in 46% of exams; when persistent OME diagnosed, tympanogram flat in 71% of exams - Tympanometry unclear/not obtained: 24% - No analytical statistics reported
Abbott et al, 2014 [50] *Cross over study*	347 children aged 0.5-6 years	GPs in primary health care, Australia	Tympanometry and pneumatic otoscopy	Standard otoscopy	- After performing tympanometry or pneumatic otoscopy, GPs were three times more likely to amend diagnosis (χ 2 = 28.64, df 1, *p* < 0.001) and management plan (χ 2 = 9.24, df 1, *p* < 0.01) made on basis of otoscopy alone - GPs preferred tympanometry, but felt cost was a barrier to routine use
Puhakka et al, 2014 [51] *Non-randomised comparative study*	600 children aged 0.6 to 14 years	Study physicians in primary health care, Finland	Tympanometry and Spectral Grade Acoustic Reflectometry (SGAR)	Pneumatic otoscopy	- Good observed agreement (86%) between SGAR and tympanometry in children - Advantages of SGAR: low cost, portability, and no need for an airtight seal - SGAR sensitivity 53% (46-59), specificity 93% (92-94), positive predictive value 48% (41-53) and negative predictive value 94% (93-95) - Tympanometry sensitivity 56% (50-62), specificity 96% (95-96), positive predictive value 60% (53-66) and negative predictive value 95% (94-96)
Alenezi et al, 2021 [52] *Non-randomised comparative study*	157 children aged 0.5-15 years	ENTs, audiologists, trained assistants at community events, Australia	Video-otoscopy images	Standard otoscopy	- Video-otoscopy images produced significantly higher quality images than traditional otoscopy, across almost all domains rated (*p* < 0.05) - Image quality significant reduced with younger patient age (*p* < 0.03)
Kleinman, K et al, 2021 [42] *Randomised controlled trial*	197 children aged 0-21 years (48% aged 0-2 years, 32% aged 3-7 years)	Paediatric emergency department and primary care clinic, US	Smartphone video-otoscopy	Standard otoscopy	- Accuracy of ear examination findings using smartphone otoscope improved by 11.2% (95% CI: 1.5, 21.8%, *p* = 0.033) relative to traditional otoscopy, to 74.8% (95% CI: 67.3, 82.1%)
**Tools for screening hearing**					
**Author/year and *study type***	**Sample**	**Study setting**	**Ref test**	**Comparator test**	**Key findings**
Newton et al. 2001 [46] *Non-randomised comparative study*	757 children aged 2.2-7.5 years. Median age 5.4 years.	Community nurses in nursery schools and child health clinics, Kenya	8-question parent/ caregiver questionnaire exploring behavioural responses to sound and communication ability designed to detect bilateral hearing loss > 40 dB HL.	ENT and audiological evaluation by ENT Clinical Officers	- 100% sensitive for bilateral hearing loss of 40 dB HL and greater and 75% specific when compared against audiometry thresholds and ENT ear observations - Negative predictive value was 100%, but positive predictive value was low, at 6.75%. - No confidence intervals reported - Authors concluded that the questionnaire, administered by healthcare workers, could be usefully applied in primary healthcare for detecting hearing impairment at the pre-school stage
Mahomed-Asmail et al. 2016 [45] *Non-randomised comparative study*	1070 children aged 5-12 years; average age 8 years.	Primary schools, South Africa	hearScreen smartphone screening app and conventional screening audiometry	Diagnostic audiometry	- No significant difference in performance - Smartphone screener and conventional screening demonstrated equivalent sensitivity (75%) and similar specificity (98.5% and 97% respectively) - Positive and negative predictive values 52.9% and 99.4% for smartphone screener, and 36.7% and 99.4% for conventional hearing screening. - No confidence intervals reported
Ramkumar et al 2018 [47] *Non-randomised comparative study*	119 children (43) and young infants (76) aged 0-5 years	Trained village health workers, community setting, India	Distortion Product Otoacoustic Emissions (DPOAE)	Tele-Auditory Brainstem Response testing	- The study found acceptable validity: sensitivity of DPOAE screening was 75% (CI: 69-81) and specificity, 91% (CI: 87-95) - Negative and positive predictive values were 99% (CI: 98-100) and 27% (CI: 21-33), respectively
Mealings et al, 2020 [44] *Non-randomised comparative study*	297 Aboriginal and Torres Strait Islander children aged 4–14 years	Primary schools, Australia	Sound Scouts game-based hearing test app for smartphones and tablets	Pure tone audiometry, *Listening in Spatialised Noise – Sentences* high-cue condition	- Sensitivity of *Sound Scouts* for average hearing loss of >20 dB HL was 41% and specificity was 89%; and for average hearing loss >30 dB HL, sensitivity at 88% and specificity at 88% - Consistent pass/fail results on *Sound Scouts* speech-in-noise measure and *Listening in Spatialised Noise – Sentences* test high-cue condition were found for 73% of children
Orzan et al. 2021 [53] *Non-randomised comparative study*	309 children aged 1-36 months	Oto-rhino-laryngology and audiology unit of a medical institute, Italy	Parental assessment of auditory skills using the Questionnaire on Hearing and Communication Abilities (QUAC)	Audiological evaluation of children at a secondary care institute	- Parents reported a decrease in auditory skills for children with sensorineural hearing loss (Χ^2^(2)=14.4, *p*=0.003), with increased concern expressed in 59% compared with 24% in normally hearing children - Positive predictive value was 0.78, but with low sensitivity (0.39) - No confidence intervals reported - Conclusion: parents have capacity to recognise non-typical auditory behaviours; an auditory abilities checklist can complement existing primary healthcare screening procedures

In the systematic review, six versions of the whispered voice test were evaluated against audiometry to determine test accuracy in detecting hearing impairment in adults and children. Sensitivity in the childhood studies ranged from 80% to 96% and specificity ranged from 90% to 98%. The systematic review authors concluded that the whispered voice test was simple and accurate but raised concerns about reproducibility, particularly in primary care settings.

The certainty of the evidence of the above studies was assessed using the Grading of Recommendations, Assessment, Development and Evaluations (GRADE) framework (VM, CK, MW, IO, SH). In general, the quality and certainty of the evidence reviewed on efficacy of common ear health and hearing check procedures (e.g., tympanometry) in primary health-like settings or by primary health-like practitioners was ‘low’ or ‘very low’, due to inadequate study designs for testing efficacy of assessments and/or the relevance of the study to the current review (e.g., small sample non-randomised studies and indirectness – such as different healthcare settings or different target populations). GRADE ratings by Ear Health and Hearing Check domains are included in Supplementary Material. The risk of bias of each included study was assessed by two independent researchers using QUADAS-2. Overall, high or unknown risk of bias was found in most studies, and particularly in studies relating to tools for screening ear health. See Supplementary Material for GRADE ratings by Ear Health and Hearing Check domain and QUADAS-2 risk of bias ratings for each included study. A limitation of the overall study evidence is that, although most studies included practitioners who may work in primary healthcare, few of the studies were carried out in primary health settings.

In addition to the 12 primary studies and reviews, eleven guidelines published between 1998 and 2020 relating to screening for otitis media and OM-related hearing loss in children were reviewed for information on components of ear health and hearing screening checks (Table 3). The quality of the reviewed guidelines was appraised using the AGREE II tool. Four researchers (VM, CK, IO, MW) independently rated the guidelines and weighted five items felt to be of most relevance to this study and came to a consensus. Weighted items were 12, 15, 18, 19, and 20 in the ‘Rigour of development’, ‘Clarity and presentation’, and ‘Applicability’ domains. Three guidelines were subsequently excluded as they did not reach a minimum average rating of 3 for the above items. Eight guidelines were included in the review: two are Australian and relate specifically to the health of Aboriginal and Torres Strait Islander people. Refer to Supplementary material for AGREE II Guidelines ratings.

**Table 3:** Guidelines included in the evidence review

**Reviewed Guidelines**
1. Otitis Media Guidelines for Aboriginal and Torres Strait Islander Children (2020) [16]
2. National guide to a preventive health assessment for Aboriginal and Torres Strait Islander people. 3 ed. (2018) [17]
3. American Academy of Pediatrics Joint Committee on Infant Hearing: Year 2007 position statement: Principles and guidelines for early hearing detection and intervention programs (2007) [54]
4. American Academy of Family Physicians, American Academy of Otolaryngology-Head and Neck Surgery, American Academy of Pediatrics Subcommittee on Otitis Media with Effusion. Otitis Media With Effusion Clinical Practice Guideline (2004) [55]
5. Clinical Practice Guideline: Otitis Media with Effusion (Update) (2016) [56]
6. American Academy of Audiology Clinical Practice Guidelines Childhood Hearing Screening (2011) [57]
7. Danish guidelines on management of otitis media in preschool children (2016) [58]
8. Korean clinical practice guidelines: otitis media in children (2012) [59]

Key findings from the evidence synthesis include:

1. Reviewed guidelines consistently recommended that parents and caregivers be asked about their concerns and their observations of their child’s ear health and hearing.
2. Undertaking both visual inspection of the ear canal and ear drum and assessing movement of the ear drum and middle ear are consistently recognised as essential to accurate evaluation of ear health status and diagnosis of otitis media in primary healthcare settings.
3. The use of scored developmental listening skills screening checklists appears to be an emerging approach of practical value to primary health practitioners and families. Results assist practitioners to infer the child’s access to sound: useful for prioritising referrals when there is limited access to audiometry, and not reflected by any other tool reviewed. Use of listening skills questionnaires can build parent/caregiver knowledge of behaviours to watch for and support, respects parents/caregivers as observers of their child, and reinforces the value of hearing to child development.
4. The role of screening audiometry in primary health ear health and hearing checks is unclear. Only two guidelines recommended inclusion. Studies found very wide variation in ability of manual pure tone audiometry screening to correctly identify presence or absence of hearing loss, which may relate to practitioner training and experience. There was little direct evidence relating to use of audiometry in such checks in primary health settings. For children aged 3 years and younger, workforce, equipment and training considerations make screening audiometry unfeasible in the context of primary health ear health and hearing checks. Automated hearing screening apps may be appropriate for older children aged 4 to 5 years.

The finding that most reviewed guidelines recommend enquiring about parent/caregiver observation led to a non-systematic citation and key-word library and Google Scholar search for studies relating to efficacy of this activity [60–65]. A review of these studies using the GRADE approach found low certainty of evidence. Although parent/carer concern (or absence) is not a good predictor of ear health and hearing status, a draft recommendation on this was put to the expert panel, as the act of asking about and following up on concerns acknowledges the importance of parent/caregiver knowledge of their children, and the primacy of their role in their child’s health and wellbeing. Additionally, when there is parent/carer concern, a proportion will be correct.

**Fig. 3:**
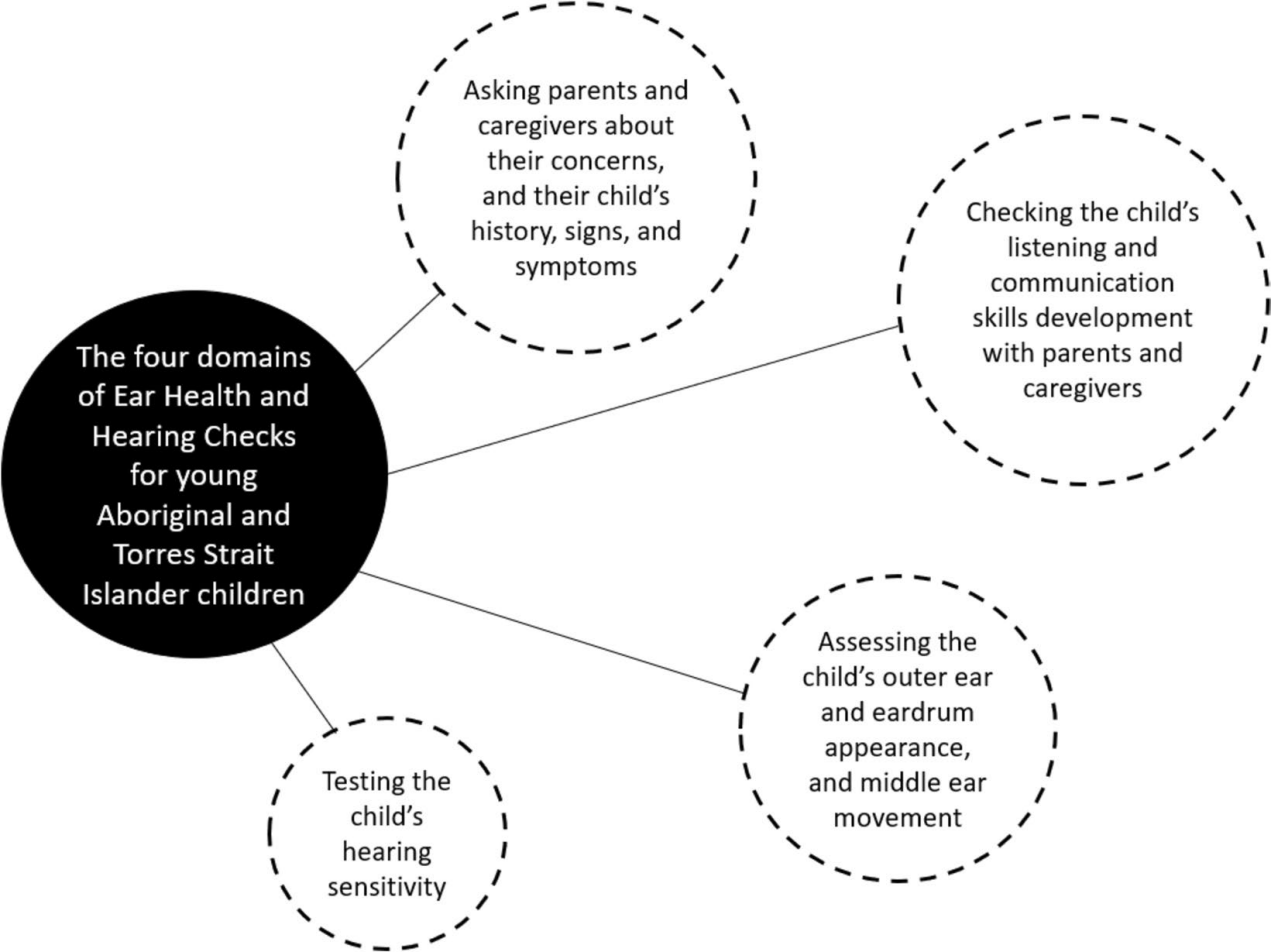
The four domains of primary health ear health and hearing checks for Aboriginal and Torres Strait Islander children aged under 6 years

The components of ear health and hearing check activities identified in the evidence review could be grouped into four domains (see Fig. 3), which then provided a structure for the draft recommendations to be put to the expert panel.

In the absence of certain and direct evidence, the perspectives and judgement of key experts on the timing of checks was particularly valued. Strong recommendations, confirming confidence that desirable effects outweigh undesired consequences, can be developed based on limited, low-quality, low-certainty evidence and a structured, expert consensus process.

### Characteristics of the expert panel

In total, 93 people were invited to the expert panel, including the 22 working group members. Of these, 79 people accepted the invitation (see Table 4). Four state/territory-level health services and nine peak bodies nominated representatives to the process. The panel included 18 Aboriginal and/or Torres Strait Islander members (23%) and 26 members (33%) working in Aboriginal and Torres Strait Islander community-controlled settings. 25% were practitioners who receive primary health referrals for children with persistent OM, including otolaryngologists, audiologists, paediatricians, and speech therapists. Four invitees were New Zealand-based, working with Māori and Pasifika communities, who experience similar ear health and hearing concerns.

**Table 4:** Characteristics of the expert panel

Expert panel characteristics	Expert panel n (%)
Gender	
Female	63 (80%)
Aboriginal and/or Torres Strait Islander	
Yes	17 (21%)
Sector^a^ - n (%)	
Primary care	47 (59%)
Secondary care	5 (6%)
Tertiary care	15 (19%)
Research	10 (13%)
Health setting	
Community controlled health service	27 (34%)
Mainstream health service	35 (44%)
Jurisdiction	
National	7 (9.9%)
New South Wales	18 (22.8%)
Queensland	12 (15.2%)
Northern Territory	11 (13.9%)
Western Australia	10 (12.7%)
South Australia	8 (10.1%)
Victoria	6 (7.6%)
Tasmania	2 (2.5%)
Australian Capital Territory	1 (1.3%)
New Zealand	4 (5.1%)
Experienced in remote settings	
Yes	29 (37%)
Profession^a^	
Aboriginal and/or Torres Strait Islander Health Worker or Health Practitioner	10 (12.7%)
Nurse (child and family health nurse or other)	17 (21.5%)
General Practitioner	8 (10.1%)
Audiologist/Audiometrist	16/1 (21.5%)
Paediatrician	3 (3.8%)
Ear Nose and Throat surgeon	7 (9.9%)

^a^Several panel members are represented in more than one category

### Findings of the e-Delphi method

**Fig. 4:**
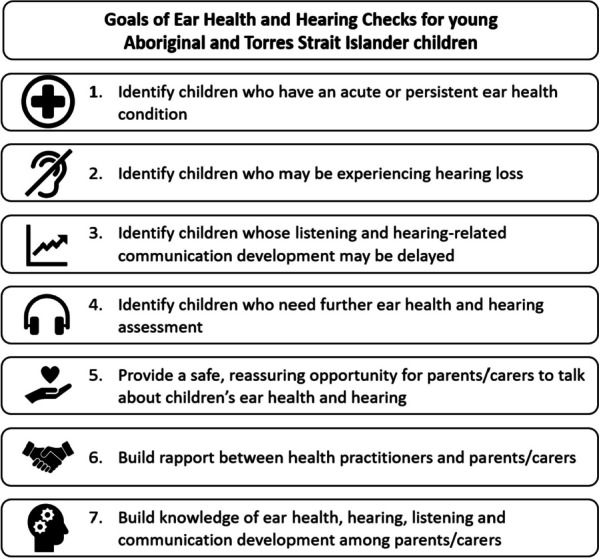
Goals of routine Ear Health and Hearing Checks for Aboriginal and Torres Strait Islander children aged under 6 years

During Round 1 of the e-Delphi survey, open during January and February 2022, sixty-five (82%) panel members took part. Seven goals of Ear Health and Hearing Checks for young Aboriginal and Torres Strait Islander children were developed by the project team and proposed to the expert panel: all reached consensus agreement in Round 1 (Fig. 4) and no further goals were identified.

Eight draft recommendations were presented to the panel, and five reached consensus agreement in the first round. In Round 2, the five recommendations that reached consensus were refined and re-presented to the panel for feedback on feasibility. Panel members were invited to comment on draft goals and recommendations in each Round. Comments were analysed thematically by members of the research team (IO, MW, SH) and the themes generated informed the refining and revising of the recommendations (refer to Supplementary Material). The three recommendations that did not reach consensus agreement in Round 1 were revised significantly and re-presented in Round 2 (open during March and April 2022). Fifty-one (65%) members of the expert panel participated in Round 2, and two more recommendations gained consensus agreement.

A recommendation relating to audiometry as part of Ear Health and Hearing Checks did not reach the pre-defined consensus level of 80%. In Round 1, a draft recommendation that ‘screening audiometry is not recommended as part of Ear Health and Hearing Checks in primary health settings for children aged three years and younger' reached 76.8% agreement, and a question on whether screening audiometry should be included in Ear Health and Hearing Checks for 4–5-year-old Aboriginal and Torres Strait Islander children reached 62.5% agreement. Analysis of invited comments found many pertained to audiometry use in broader primary healthcare, and not specifically to its use within routine Ear Health and Hearing Checks. Responses identified by the research team that specifically related to inclusion of audiometry within the Checks did not support such use. Analysed themes related to training and skill maintenance, time constraints, access to equipment, a lack of need to include audiometry (as other components can indicate when audiometry may be required), and the challenges and risks associated with primary health practitioners undertaking and interpreting audiometry with children aged under 5 years. In Round 2, the draft audiometry recommendation was substantially revised to ‘As part of routine Ear Health and Hearing Checks in primary health settings, audiometry is not recommended’. Once again, the pre-determined 80% consensus level was not reached (the result was 71%). Thematic analysis of comments identified some recurring themes from Round 1, as well as new themes including the limitations of single-instance audiometry for assessing children with OM, the need for clinical governance of audiometry, and that audiometry should only be required when there are indications of persistent or recurrent OM or hearing impairment. Analysis of comments made by 7 of 12 respondents who felt audiometry should be part of routine Checks identified a common theme supporting inclusion of audiometry under certain conditions: when concerns arise during the Check, and prior to starting school. After two Working Group discussions, a final consensus recommendation on the role of audiometry in the Checks could not be made. However, a general recommendation on the role of audiometry for Aboriginal and Torres Strait Islander children with OM, aligning with the *Otitis Media Guidelines for Aboriginal and Torres Strait Islander Children* [16] was agreed upon*.*

### Final ear health and hearing check recommendations

The eight finalised recommendations outline the agreed goals, components, and timing of primary health Ear Health and Hearing Checks for Aboriginal and Torres Strait Islander aged under 6 years (Table 5). Recommendations 1, 2, 3, and 7 relating to parent/carer report, listening skills, assessment of appearance and movement of the middle ear, and timing of the checks are classified as strong despite low certainty of evidence. These discordant recommendations, where strong recommendations are formed in the absence of high-quality evidence, were each felt to be justified in view of the known high prevalence and harms of asymptomatic, undetected/untreated, persistent OM in the target population [9, 11–13, 15, 66, 67], and the high level of agreement from expert panel members, as well as meeting the GRADE criteria for a strong recommendation despite low quality evidence [68]. For recommendation 1 (asking about parent/carer concern), although the evidence relating to the benefits of asking parents/carers about signs and symptoms was uncertain, the risk of missing a persistent hearing problem, potentially leading to developmental harm, and the value of affirming parental/carer centrality in the child’s life, warranted a strong recommendation. In relation to recommendation 2, there is currently little evidence associated with the benefits of screening listening skills as part of routine primary health ear health and hearing checks; however, no other component of the check has the potential to differentiate OM persistence and flag possible auditory deprivation and associated developmental risk in isolation of the child’s clinical history. For recommendation 3 (assessing appearance and mobility of the middle ear), high certainty evidence exists for the use of pneumatic otoscopy and for tympanometry for detecting otitis media with effusion, including in primary health settings, for populations not known to experience high rates of persistent ear disease. Although there is very little direct evidence from Aboriginal and Torres Strait Islander primary health settings, given high certainty of similar benefits, a strong recommendation was formed relating to use of *either* simple otoscopy plus tympanometry *or* pneumatic otoscopy. In relation to recommendation 7 (the timing of checks), relative to current recommendations to check ears at *every* primary health visit, there is little-to-no evidence relevant to the benefits of either approach. However, the consensus opinion was that a pre-determined schedule of checks that reflected the natural history of OM for Aboriginal and Torres Strait Islander children and the sensitive period for development of children’s listening and communication skills would be less risky than checks undertaken at indeterminate intervals, and therefore warranted a strong recommendation.

**Table 5:** Final Ear Health and Hearing Check recommendations presented with GRADE rating of certainty of evidence, ratings of expert agreement and feasibility, and strength of recommendations

		Strength of recommendation	Certainty of evidence	Level of expert agreement	Expert feasibility rating
Domain: Parent and carer-reported history, concerns, signs, and symptoms					
1.	Ask parents/carers about: a) their child’s ear health (recent and longer term); b) any concerns about their child’s ear health, hearing, or communication	Strong	Low	96%	92%
Domain: Listening and communication skills					
2.	From the age of six months, review children’s listening and communication skills development with parents/carers using appropriate questionnaires or checklists	Strong	Very low	98%	88%
Domain: Ear health					
3.	Examine appearance of the ear canal and ear drum, and assess movement of the ear drum and middle ear using either simple otoscopy plus tympanometry OR pneumatic otoscopy	Strong	Low	93%	82%
4.	Use of video otoscopy is suggested for health promotion purposes with parents/carers, and/or for sharing images with other healthcare practitioners	Conditional	Low	96%	71%
Domain: Hearing sensitivity					
5.	Otoacoustic emissions (OAE) testing is suggested to confirm or exclude normal or near-normal hearing when: - equipment is available - primary health practitioners have capability and confidence to use it - there is a local preference for using OAE testing	Conditional	Low	84%	75%
6.	Audiometry is recommended as per *Otitis Media Guidelines *[16] when: - there are parent/carer and/or practitioner concerns about ear health, hearing, or communication, and/or - the child’s listening and communication development are not yet on track, and/or - there is a persistent or recurrent middle ear condition	Strong	--	--	--
Timing of routine Ear Health and Hearing Checks					
7.	Following newborn hearing screening, Ear Health and Hearing Checks are recommended at least 6 monthly until the age of 4 years, and then one check at 5 years old	Strong	Low	88%	67%
8.	It is suggested that Ear Health and Hearing Checks be undertaken more frequently than 6 months: - in high-risk settings, and/or - for children aged under two years, and/or - when it is acceptable to families, and/or - in response to parent/carer concerns	Conditional	Low	88%	64%

Three recommendations (4, 5, and 8) are conditional, or relevant in certain circumstances, including those relating to the use of video-otoscopy and otoacoustic emissions, and when more frequent checks may be required. These are outlined in Table 5 and the companion paper provides further detail on the recommendations [23].

## Discussion

This project used a rigorous, appropriate, and culturally sensitive process to meet the need for trustworthy and effective recommendations that support consistent, routine Ear Health and Hearing Checks for Aboriginal and Torres Strait Islander children in primary health care settings. Aboriginal and Torres Strait Islander practitioners and researchers were members of the project team, working group, and expert panel, embedding cultural sensitivity and community control of research throughout the project. There was substantial involvement of practitioners and managers from primary health settings in the process, and formal participation from a range of health departments, services, and peak bodies.

While a level of agreement was reached on the role of audiometry in routine Ear Health and Hearing Checks, this did not reach pre-determined consensus levels (80%). As a recommendation could not be formed, the working group and project team elected to reiterate the audiometry recommendation made in the OM Guidelines [16].

A limitation of this study was that the input of parents and caregivers of Aboriginal and Torres Strait Islander who have experienced persistent OM in early childhood was not explicitly sought; however, among the working group and expert panel members were parents or caregivers of children who have experienced this condition and the range of negative impacts on both the child and their family.

Although this project has developed strong recommendations through an evidence review and e-Delphi consensus process, barriers to implementation will need to be addressed, and sustainable resourcing identified for existing and new strategies to support implementation. Many studies, particularly that address Aboriginal and Torres Strait Islander health issues, produce evidence-based findings, but do not translate into improved policy, practice, and health outcomes. High-quality evidence produced through a research process that engages effectively with policymakers is more likely to result in translation into practice [69]. This project has achieved this to some extent through involvement of policymakers in the consensus process, through provision of early feedback on project findings, and through inviting input on implementation of the recommendations into practice in primary health care. A companion paper is available that provides a detailed overview of the recommendations and summarises barriers to implementation cited by key experts during the project [23]. This body of work provides Aboriginal and Torres Strait Islander and mainstream primary health services with best available information on when and how to check young Aboriginal and Torres Strait Islander children’s ear health and hearing. Although some services may be well-placed to commence implementation, a follow-on project to scope implementation is needed, to understand what it would take for the recommended Checks to be incorporated into routine clinical practice in the settings young Aboriginal and Torres Strait Islander children and their families access primary care. The scoping would need to consider implementation in ways that meet the needs of their community and service, are supported by partnerships and collaboration (including for accessing referral services), and align with Continuous Quality Improvement principles [70].

## Conclusions

In the absence of rapid improvement in the social and environmental factors that lead to ear infections in more than one in three [1, 2] Aboriginal and Torres Strait Islander children, it is imperative that we succeed at identifying persistent ear and hearing problems in children’s first years of life and provide effective, holistic care to the child, and clear, practical information to their family. To assist with early identification, we developed strong recommendations for ear health and hearing checks through a systematic scoping review and a large, national e-Delphi consensus process. The project was guided, culturally, clinically, and technically, by Aboriginal and Torres Strait Islander and non-Indigenous experts in hearing, healthcare, and research with Aboriginal and Torres Strait Islander people and communities. The process involved substantial representation of key stakeholders involved in providing ear health and hearing checks. The recommendations address a gap in existing clinical guidelines for management of OM in Aboriginal and Torres Strait Islander children. The development process adhered to the principles of ethical research in Aboriginal and Torres Strait Islander health and may be applicable to other primary healthcare checks relevant to Aboriginal and Torres Strait Islander children and adults. Acknowledging the current resource challenges facing Australian primary healthcare, we urge practitioners, services, and systems to embed six-monthly ear health and hearing checks into routine care during the first years of Aboriginal and Torres Strait Islander children’s lives.

### Supplementary Information

**Supplementary Material 1.** 

## Data Availability

The datasets used and/or analysed during the current study are available from the corresponding author on reasonable request.

## Abbreviations

OM: Otitis Media
OME: Otitis Media with Effusion
EHHC: Ear Health and Hearing Check
ENT: Ear, Nose and Throat
GRADE: Grading of Recommendations, Assessment, Development and Evaluations
SGAR: Spectral Gradient Acoustic Reflectometry
