# Supplementary material for: Development of the national consensus statement on ear health and hearing check recommendations for Aboriginal and Torres Strait Islander children aged under 6 years attending primary care: systematic scoping review and e-Delphi
Source: BMC Prim Care. 2024 Mar 14;25:86. doi: 10.1186/s12875-024-02307-6 (PMC10938761; doi:10.1186/s12875-024-02307-6)
Supplement: Supplementary file 1 — Supplementary Material 1. [file 12875_2024_2307_MOESM1_ESM.pdf]

Supplementary materials

Ear Health and Hearing Check key expert consultation: Report on results of Round 1 survey **2**

Introduction 2

Proposed goals of routine Ear Health and Hearing Checks 4

Domain 1: Parent/carer-reported history and concerns 6

Domain 2: Listening and communication skills 8

Domain 3: Ear health 10

Domain 4: Hearing sensitivity 13

Timing or scheduling of checks 20

Overview of outcomes of Round 2 of the Ear Health and Hearing Check key expert consultation **25**

Foreword 25

Participants in the Round 2 consultation 25

Proposed goals of routine Ear Health and Hearing Checks 26

Domain 1: Parent and carer-reported history, concerns, signs, and symptoms 26

Domain 2: Listening and communication skills 27

Domain 3: Ear health 28

Domain 4: Hearing sensitivity 29

Timing of Ear Health and Hearing Checks 30

Audiometry 33

Search strategies **34**

Database: EBM Reviews - Cochrane Database of Systematic Reviews <2005 to July 14, 2021> 34

Scopus Advanced search 38

Database: Ovid MEDLINE(R) ALL <1946 to July 09, 2021> 39

Informit Advanced search 44

Database: Embase Classic <1947 to 1973>, Embase <1974 to 2021 July 09> 45

CINAHL Complete Search string 50

Database: EBM Reviews - Cochrane Central Register of Controlled Trials <June 2021> 55

QUADAS-2 risk of bias ratings **59**

Agree II Guidelines ratings **60**

GRADE ratings by Ear Health and Hearing Check domain **7**

Ear Health and Hearing Check key expert consultation: Report on results of Round 1 survey

Introduction

In the first round of the Ear Health and Hearing Check survey consultation, 65 people took part: 83% of the key expert group. Respondents reported working in urban, regional, and remote areas, in a roughly even spread. Most reported working in primary health services, just over half in community-controlled settings.

Three of the draft recommendations presented to the expert panel reached consensus, that is, a level where more than 80% of survey respondents agreed with the recommendation.

In the lead-up to the second round of the survey, substantial revisions have been made to the recommendations that did not reach consensus (those relating to audiometry, otoacoustic emissions, and timing), and minor revisions made to the other recommendations.

On feasibility

Information gathered on feasibility in the Round 1 survey has influenced the recommendations being presented in Round 2. As substantial revisions have been made to recommendations relating to both components and timing of the Ear Health and Hearing Checks since Round 1, the survey will again ask about the feasibility of the draft recommendations. The levels of consensus and comments on feasibility from both Rounds of the consultation will guide further work around implementation of the recommendations after this project. It is important to note that expert opinion on feasibility is shaping the recommendations, and that the final recommendations can be agreed on before consensus on feasibility is achieved.

In this round, three revised recommendations will be presented, and questions about feasibility for all eight draft recommendations will be asked. Table 1 below presents the proposed draft recommendations.

On 'routine Ear Health and Hearing Checks'

The routine Ear Health and Hearing Checks these recommendations refer to are intended to be undertaken by primary healthcare practitioners as part of standard care, whether parents/carers have expressed concern about their child's ear health or hearing, or not.

> As a reminder, this activity relates to the <u>components</u> and <u>timing</u> of routine Ear Health and Hearing Checks for:
>
> - Aboriginal and Torres Strait Islander children
> - aged under 6 years
> - attending primary healthcare settings, and
> - who are <u>not</u> already being treated for ear health and/or hearing problems because:
>   - they are not known to have OM and/or hearing loss, or
>   - they have been recognised as having OM and/or hearing loss but are not in active care.

It is critical that these recommendations provide primary health practitioners with sufficient information to decide whether a child needs further assessment, triaging for referral, or close monitoring.

On the important role of key experts in this process

It is important to keep in mind that the project team found little high-quality evidence from published studies about the efficacy (how well a test performs in controlled conditions) of certain ear health and hearing procedures in primary health settings. Because of this, expert input and consensus-building is particularly important in developing these recommendations, so that primary healthcare practitioners can feel confident that the recommendations are robust and trustworthy.

It is also important to remember that strong recommendations can be made through the building of expert consensus, even when high-quality published evidence is lacking.

Overview of recommendations being presented in Round 2.

The following table (Table 1) shows the revised draft recommendations being presented in this round.

Recommendation	Proposed strength of recommendation	Overall evidence quality
**Domain 1: Parent and carer-reported history, concerns, signs and symptoms**		
Draft recommendation 1:<br>As part of routine Ear Health and Hearing Checks in primary health care settings, ask parents/carers about: 1) their child's ear health (recent and longer term); 2) any concerns about their child's ear health, hearing, or communication.	To be confirmed (TBC)	(◯◯◯◯)
**Domain 2: Listening and communication skills**		
Draft recommendation 2*:*<br>As part of routine Ear Health and Hearing Checks in primary health care settings, from the age of six months, review children's listening and communication skills development with parents/carers using appropriate questionnaires or checklists.	TBC	(⊕◯◯◯)<br>Very Low
**Domain 3: Ear health**		
Draft recommendation 3*:*<br>As part of routine Ear Health and Hearing Checks in primary health care settings, examine the appearance of the ear canal and ear drum, and assess the movement of the ear drum and middle ear using either simple otoscopy plus tympanometry OR pneumatic otoscopy.	TBC	(⊕◯◯◯)<br>Very Low
Draft (conditional) recommendation 4:<br>As part of routine Ear Health and Hearing Checks in primary health settings, use of video otoscopy is suggested for health promotion purposes with parents/carers, and/or for sharing images with other health care practitioners.	TBC	
**Domain 4: Hearing sensitivity**		
Draft recommendation 5**:**<br>As part of routine Ear Health and Hearing Checks in primary health care settings, audiometry is not recommended.	TBC	(◯◯◯◯)<br>No evidence found
Draft (conditional) recommendation 6:<br>As part of routine Ear Health and Hearing Checks in primary health care settings, otoacoustic emissions (OAE) testing is suggested to confirm or exclude normal or near-normal hearing when:<br>– equipment is available<br>– primary health practitioners have capability and are confident to use it<br>– there is a local preference for using OAE testing.	TBC	(⊕⊕◯◯)<br>Low
**Timing of Ear Health and Hearing Checks**		
Draft recommendation 7:<br>Following newborn hearing screening, Ear Health and Hearing Checks are recommended at least 6 monthly until the age of 4 years, and then one check at 5 years old.	TBC	
Draft (conditional) recommendation 8:<br>It is suggested that Ear Health and Hearing Checks be undertaken more frequently than 6 months:<br>– in high-risk settings, and/or<br>– for children aged under two years, and/or<br>– when it is acceptable to families, and/or<br>– in response to parent/carer concerns.	TBC	

Table 1: Summary of the current revised recommendations

Brief overview of changes to recommendations

Consensus was not reached on the round 1 draft recommendations relating to audiometry, otoacoustic emissions (OAEs), and timing.

Taking into consideration the lack of consensus and the comments from the expert panel in the first survey, substantial changes have been made to these recommendations. The details and rationale for these revisions are provided later in this survey.

The remaining Round 1 draft recommendations on parent/carer-reported history and concerns, listening and communication skills, examining appearance and movement, and video otoscopy all reached consensus, but some minor revisions have been made to each of these.

Changes to the recommendations on Timing may influence perspectives on the feasibility of recommendations, therefore this survey asks again about the feasibility of each recommendation.

Figure 1 below provides an overview of the recommended components and timing of routine Ear Health and Hearing Checks in primary health settings being presented for consideration in Round 2 of this consultation process:

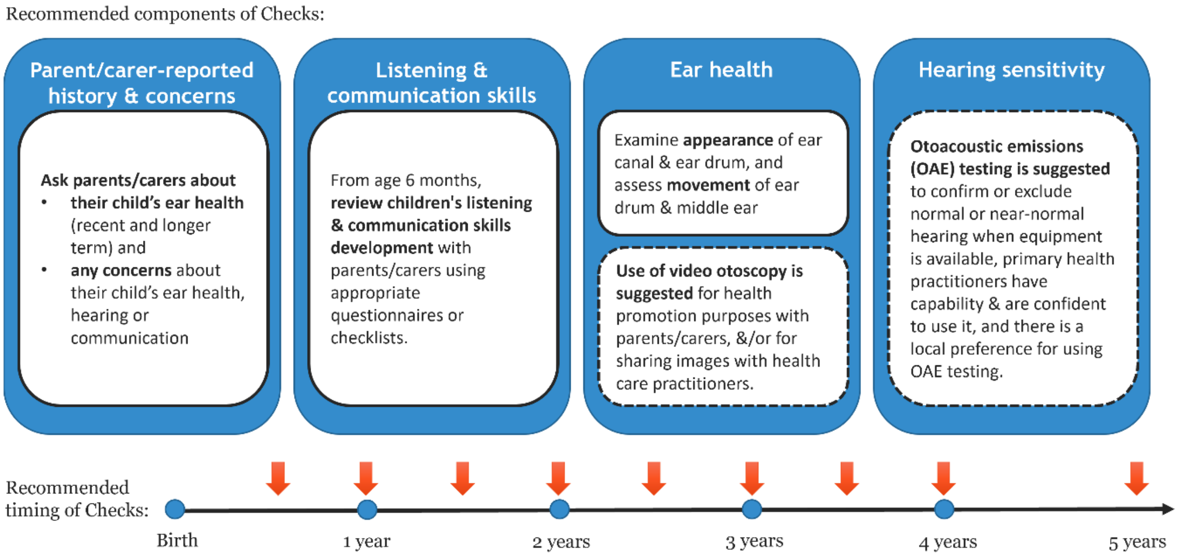

Figure 1: Proposed components and timing of routine Ear Health and Hearing Checks

Proposed goals of routine Ear Health and Hearing Checks

Round 1 draft recommendation

In round 1, the following goals of routine Ear Health and Hearing Checks were proposed:

1. *Identify children who have good ear health, hearing, and listening and communication development*
2. *Identify children who have an acute or persistent ear health condition*
3. *Identify children who may be experiencing hearing loss*
4. *Identify children whose listening and hearing-related communication development may be delayed*
5. *Identify children who need further ear health and hearing assessment*
6. *Provide an opportunity for parents/carers to talk about children's ear health and hearing*
7. *Build rapport between health practitioners and parents/carers*
8. *Build knowledge of ear health, hearing, listening and communication development among parents/carers.*

These questions were asked:

a) How important are each of these as goals of Ear Health and Hearing Checks?
b) Would you add other goals to these?

Summary of key expert responses

- Consensus was reached for each of the goals (between 86.15 to 100% agreement).

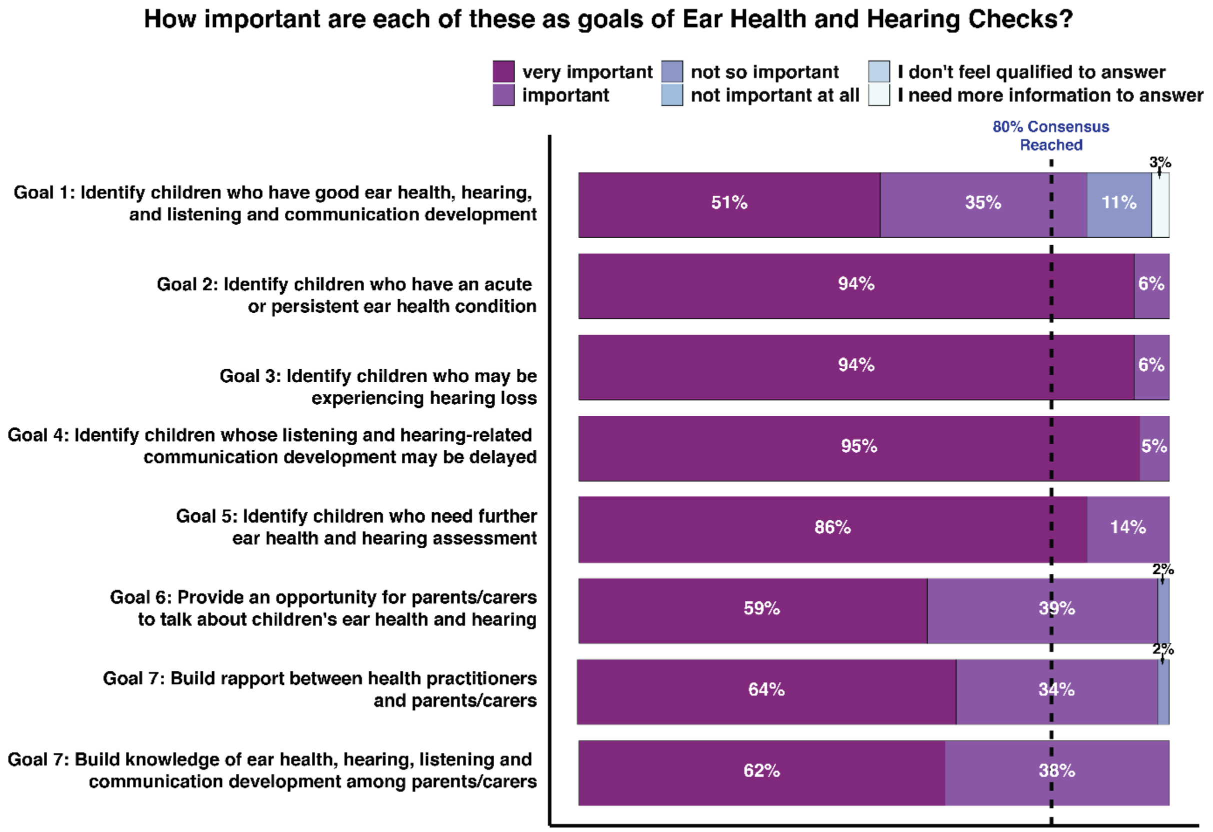

Comments by key experts

A diverse range of additional goals or ideas were suggested by respondents. Broadly they fell into the following themes:

- Build parent/carer knowledge about ear health and hearing (including signs, prevention, importance of hearing and impacts of OM)
- Empower families with knowledge, skills, resources and contacts to support their child's ear health and hearing needs
- Enable good data collection and reporting
- Identify and document children at risk due to family history of OM
- Identify children who need referrals and support for communication development issues
- Improve cultural safety in ear and hearing healthcare
- Monitor/evaluate the implementation of checks
- Promote children understanding and talking about their ear health and hearing
- Promote strong health literacy in communities
- Provide capacity building for primary health workers
- Provide clear referral pathways
- Provide streamlined support, access, and advocacy for families to ensure timely follow-up and care
- Addressing wider social determinants of OM needed

While they are all valuable comments that inform future discussions on implementation of the checks, the project team has not proposed any additional goals in round 2 of the survey.

Outcome

No changes were made to the goals of routine Ear Health and Hearing Checks proposed in Round 1.

Domain 1: Parent/carer-reported history and concerns

Round 1 draft recommendation

In Round 1, the following draft recommendation was proposed:

Draft recommendation 1: Ask parents/carers about: 1) their child's ear health over the past three months; 2) any concerns about their child's ear health, hearing, or communication.

These questions were asked:

a) How strongly do you agree with this draft recommendation?
b) How strongly do you agree with the time period of 3 months when asking parents/carers about their child's ear health history?
c) How feasible is it for this recommendation to be implemented in primary health settings?
d) Comments

Summary of key expert responses

– 96% of respondents agreed with the draft recommendation.

– 89% agreed with the time period of 3 months. 84% of respondents found this feasible.

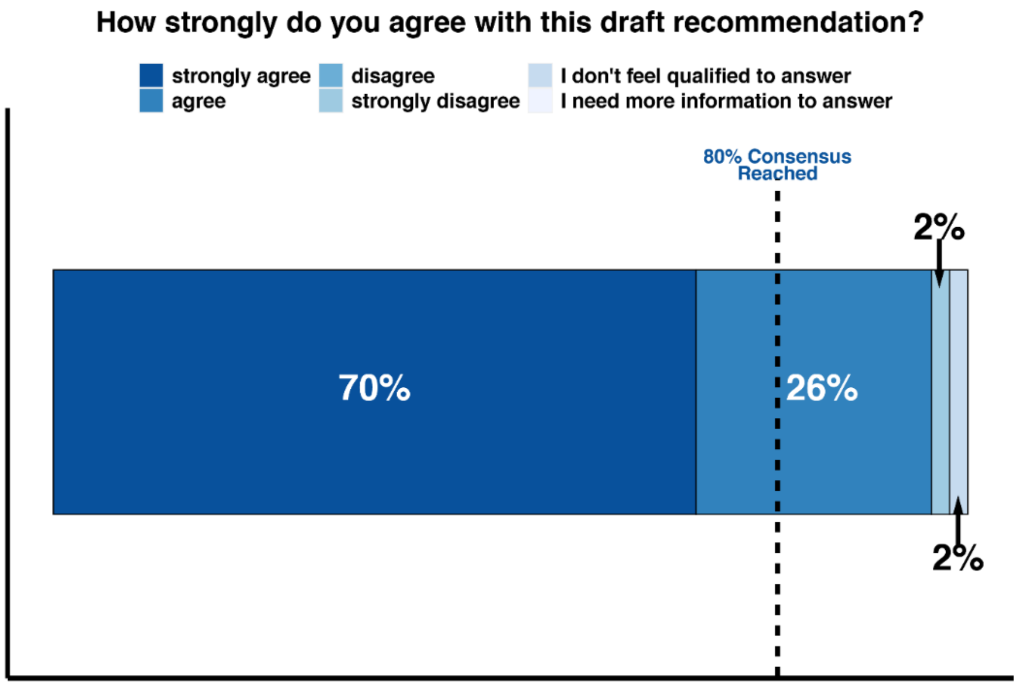

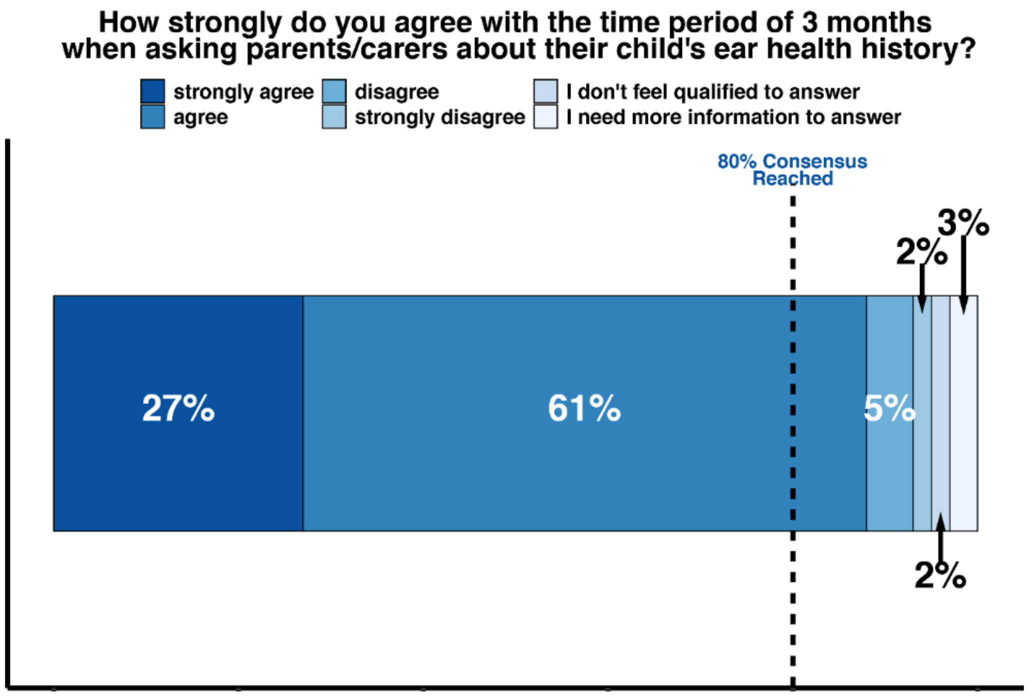

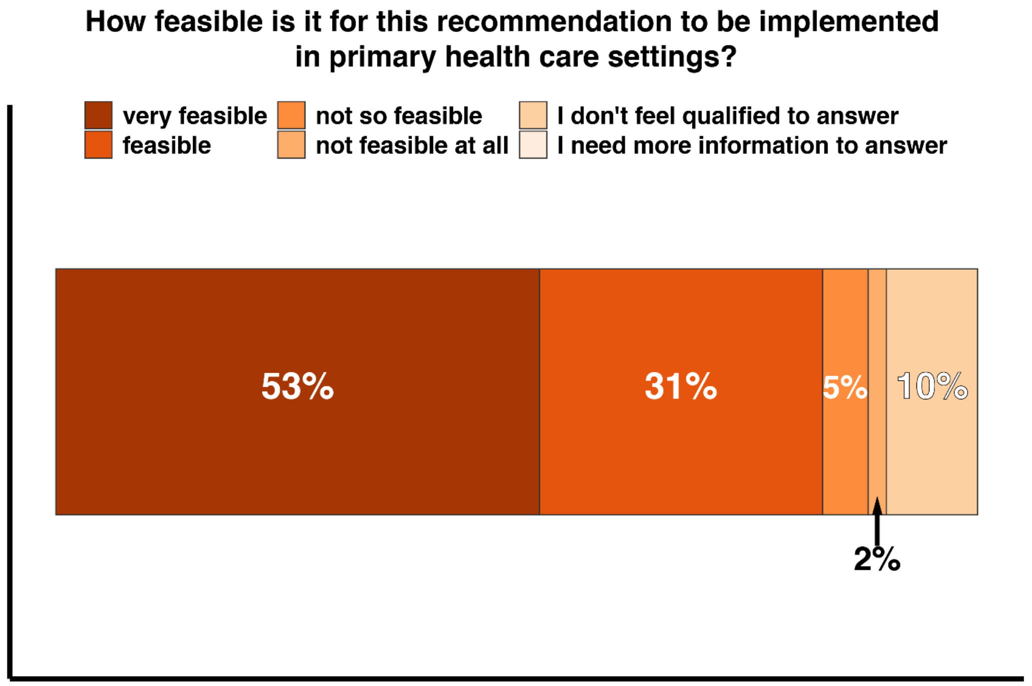

Themes of comments

- caregivers may find it challenging to respond to questions about concerns and 3-month history
- asking about the history should not be restricted to 3 months.
- asking caregivers about concerns has benefits for families and health workers (for awareness/education)
- caregivers may find it challenging to respond to questions due to different caregivers
- parents not reporting concerns/engaging as worried about being judged
- more structure for asking caregivers about concerns/history is useful/needed
- needs to be built into health systems and recall databases
- strengths-based questions are also needed
- time constraints

– training and resources are needed

Round 2 revised draft recommendation

Taking into consideration the expert panel's comments, this recommendation has been revised from asking parents/carers about their child's ear health over the last three months to asking about their child's recent and longer-term ear health. It is important to understand whether ear health problems are transient, recurrent, or persistent. Revising the wording to a less specific time period may increase feasibility and acceptability of the recommendation.

The proposed final recommendation is:

Draft recommendation 1: As part of routine Ear Health and Hearing Checks in primary health settings, ask parents/carers about: 1) their child's ear health (recent and longer term); 2) any concerns about their child's ear health, hearing, or communication.

Domain 2: Listening and communication skills

In Round 1, the following draft recommendation was proposed:

Draft recommendation 2: From age six months, as part of Ear Health and Hearing Checks, review children's listening and communication skills development with parents/carers using appropriate questionnaires or checklists, at scheduled points* and when concerns arise.

**The draft recommendation on timing of Checks proposes listening and communication skills reviews at, at least, three monthly until 24 months of age (a combination of 'minimum' and 'full' checks), twice while aged two years, and annually at age three, four and five years.*

These questions were asked:

a) How strongly do you agree with this draft recommendation?
b) How feasible is it for this recommendation to be implemented in primary health settings?
c) Any comments?

Summary of key expert responses

– Consensus was reached: 98.3% of respondents agreed with this draft recommendation, and 76.7% felt it was feasible to implement in primary health settings.

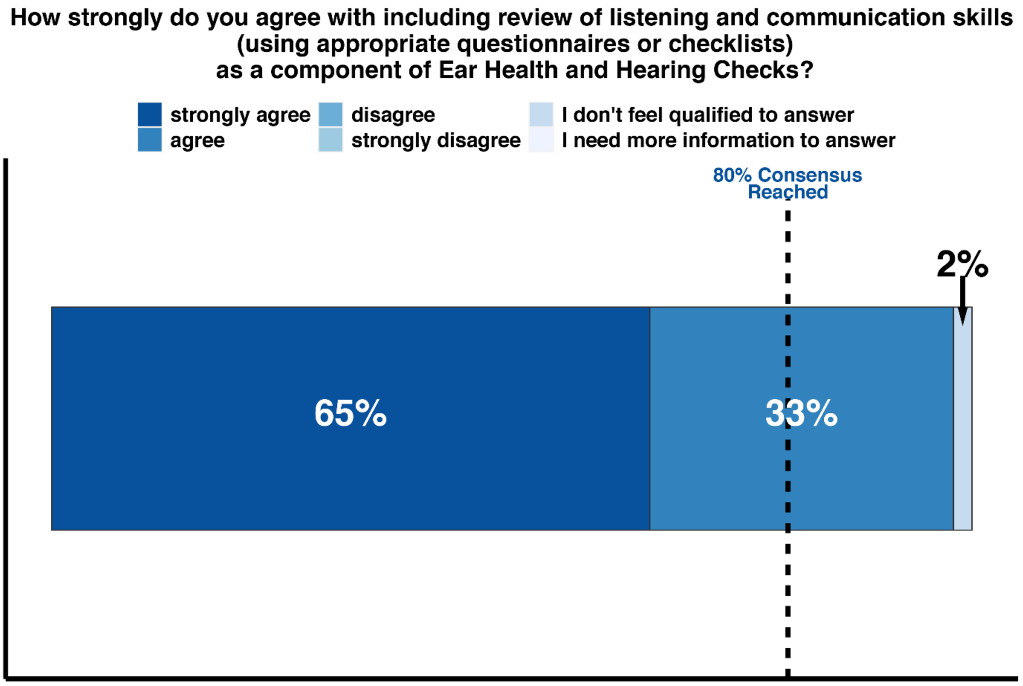

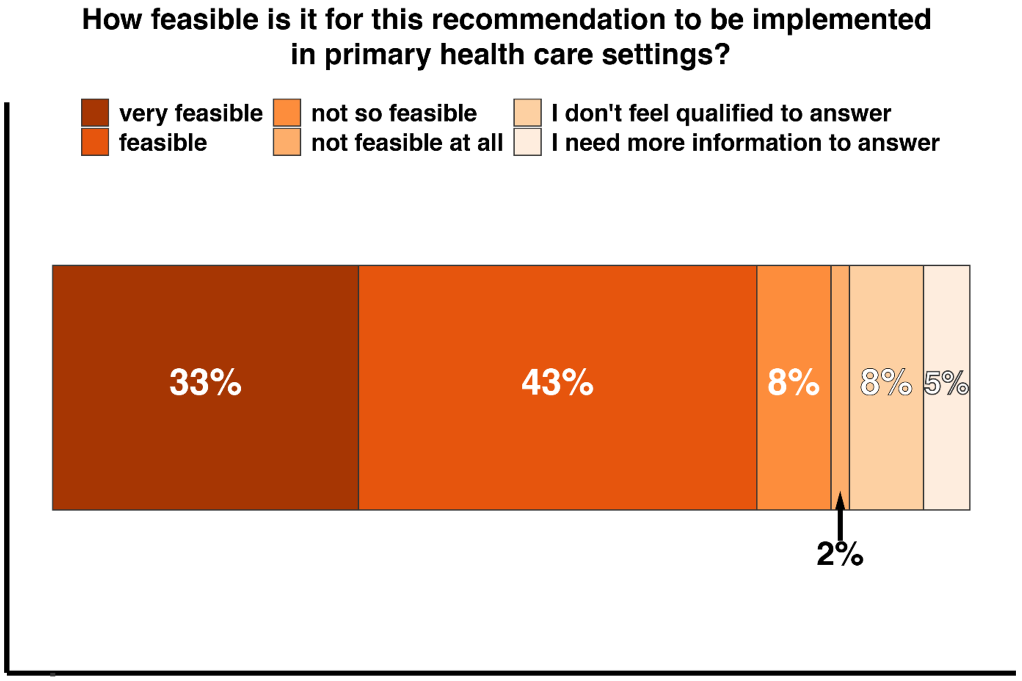

Themes of comments

- accessible referral pathways and evaluation needed
- needs to be built into health systems and recall databases
- questionnaires need to be culturally and linguistically appropriate
- questionnaires have benefits for families and health workers
- rebates/funding needed
- resources, support, and training for primary healthcare workers needed
- time constraints/competing priorities

Round 2 revised draft recommendation

The project team proposes a minor revision to this recommendation, leaving out the part 'and when concerns arise'. This will now be covered in the draft timing recommendations. It is important to note that other changes made to proposed timing and components of the Checks may increase overall feasibility of this recommendation.

The proposed final recommendation is:

Draft recommendation 2: As part of routine Ear Health and Hearing Checks in primary health settings, from the age of six months, review children's listening and communication skills development with parents/carers using appropriate questionnaires or checklists.

Domain 3: Ear health

Round 1 draft recommendation 3a

In Round 1, two draft recommendations relating to Ear Health were proposed, the first one being the following:

Draft recommendation 3a: Examine the appearance of the ear canal and ear drum and assess the movement of the ear drum and middle ear using either simple (video) otoscopy plus tympanometry OR pneumatic (video) otoscopy.

Draft recommendation 3b: Consider use of video otoscopy for health promotion purposes with parents/carers and/or for sharing images for the purposes of specialist ear health care.

These questions were asked:

Draft recommendation 3a

a) How strongly do you agree with examining appearance as part of routine Ear Health and Hearing Checks?
b) How strongly do you agree with assessing movement as part of routine Ear Health and Hearing Check?
c) How strongly do you agree with assessing BOTH movement and appearance as part of routine Ear Health and Hearing Checks?
d) How feasible is it for this recommendation to be implemented in primary health settings?
e) Any comments?

Draft recommendation 3b

a) How strongly do you agree with the recommendation?
b) How feasible is it for this recommendation to be implemented in primary health settings?
c) Any comments?

Key expert responses:

Draft recommendation 3a

– 97% of respondents agreed with examining the appearance of the ear drum, and 91% agreed with examining the movement of the ear drum.
– 93% agreed with examining both movement and appearance of the eardrum as part of routine Ear Health and Hearing checks
– 67% agreed that the recommendation was feasible to implement.

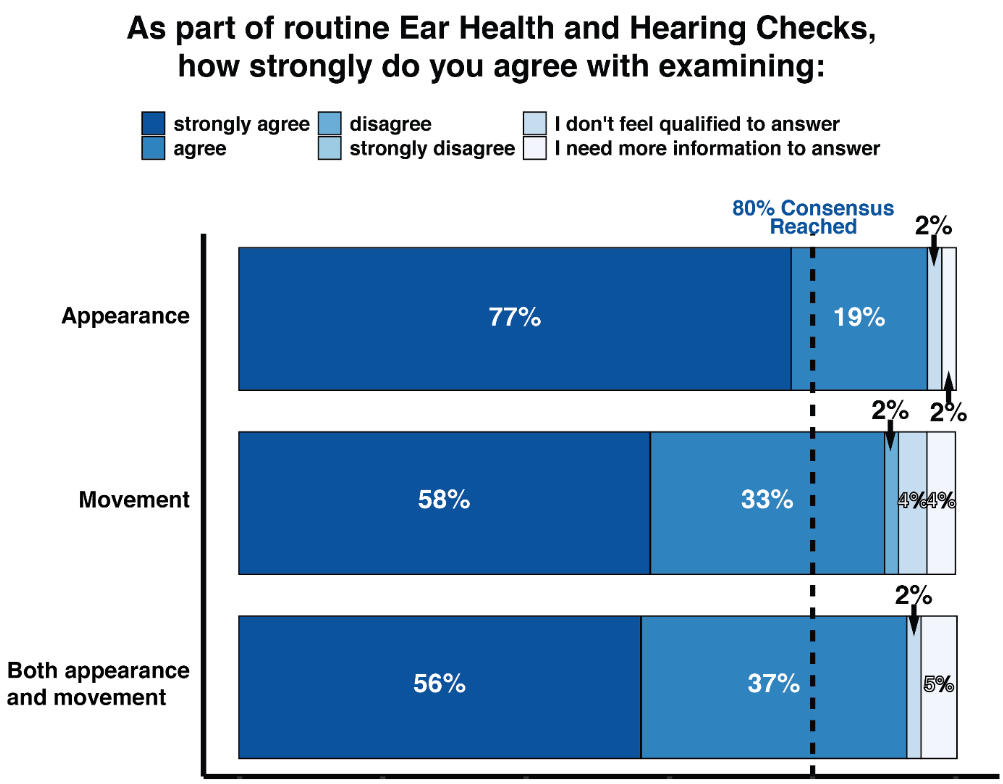

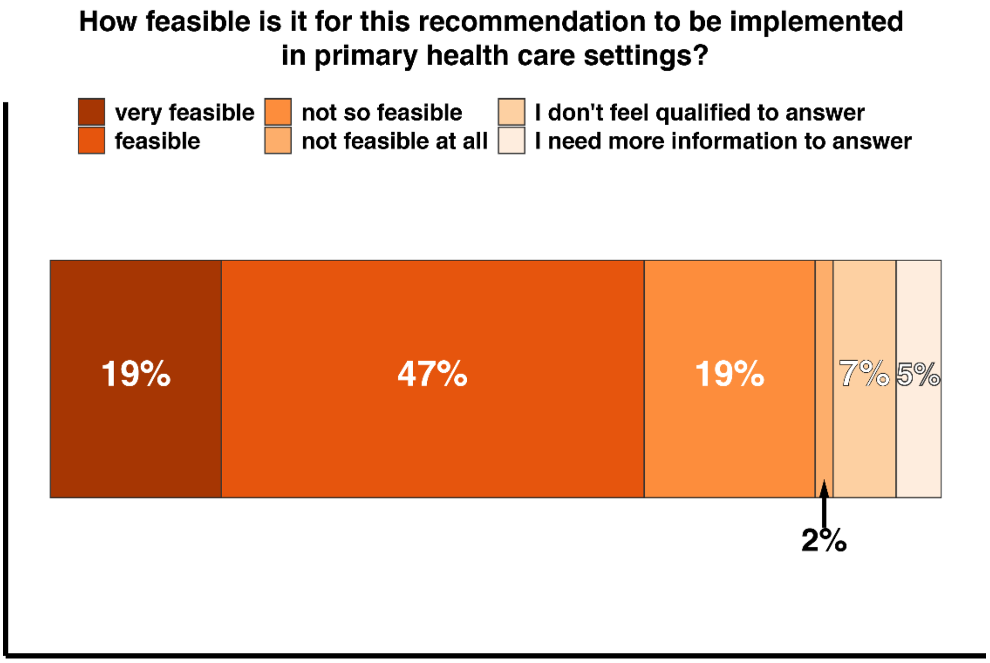

Draft recommendation 3b

- 96% agreed with the recommendation to consider use of video otoscopy as part of Ear Health and Hearing Checks
- 67% agreed that the recommendation was feasible to implement.

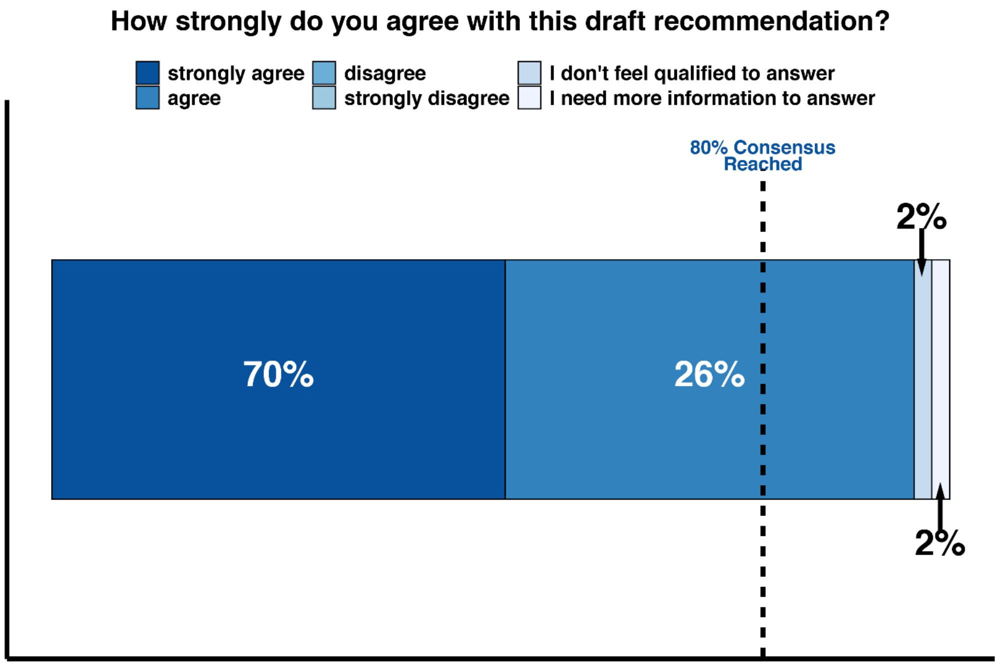

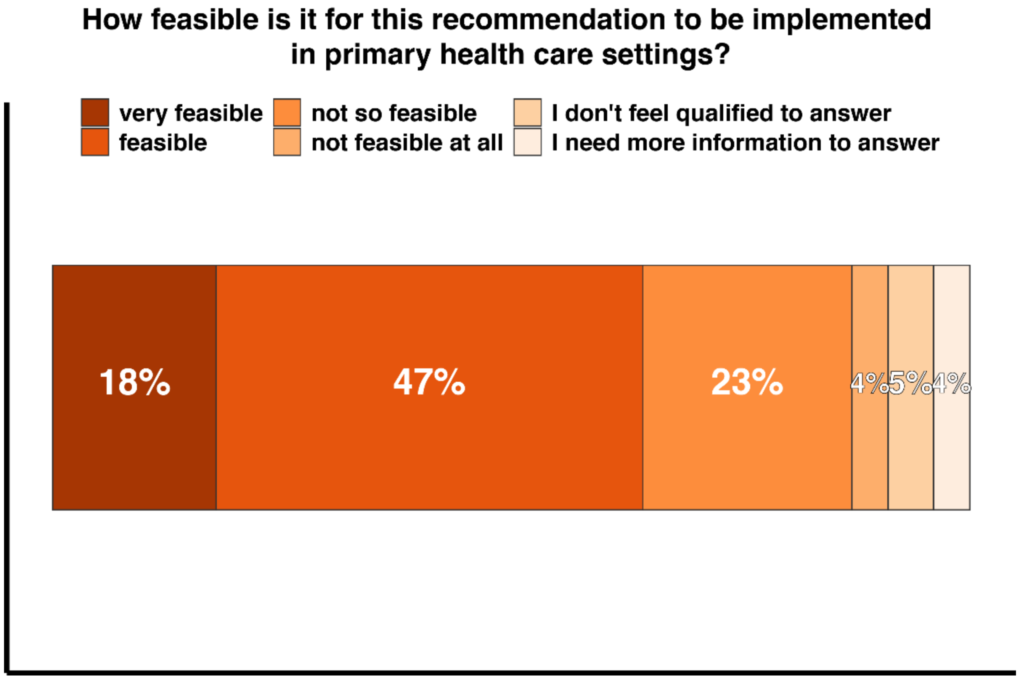

Themes of the comments:

Draft recommendation 3a

- equipment and maintenance access/costs/burden of comments
- needs to be built into health systems and recall databases
- pneumatic otoscopy has risks
- rebates/funding needed
- resources, support, and training for PHC workers needed
- time constraints/competing priorities
- tympanometry is preferable to pneumatic otoscopy

– vital to assess both movement and appearance

Draft recommendation 3b

– video otoscopy has benefits for health promotion and engagement
– video otoscopy has benefits for clinical management
– time constraints/competing priorities
– resources, support, and training for PHC workers needed
– referral pathways needed
– equipment and maintenance access/costs/burden
– rebates/funding needed
– needs to be built into health systems and recall databases

Round 2 revised draft recommendations

Draft recommendation 3a

– A minor revision was made to this recommendation: '*As part of routine Ear Health and Hearing Checks in primary health settings…'* was added to the beginning, to make the context of the recommendation clear.

Draft recommendation 3: As part of routine Ear Health and Hearing Checks in primary health settings, examine the appearance of the ear canal and ear drum, and assess the movement of the ear drum and middle ear using either simple otoscopy plus tympanometry OR pneumatic otoscopy.

Draft recommendation 3b

Minor revisions were made to the draft recommendation:

– 'Consider use of video otoscopy…' was replaced with 'use of video otoscopy is suggested…'
– Further, *'As part of routine Ear Health and Hearing Checks in primary health settings'* was added, to make the context of the recommendation clear.
– Finally, the recommendation was re-numbered from '3a' to '4'.

Draft (conditional) recommendation 4: As part of routine Ear Health and Hearing Checks in primary health settings, use of video otoscopy is suggested for health promotion purposes with parents/carers, and/or for sharing images with other health care practitioners.

Domain 4: Hearing sensitivity

4.1 Audiometry in routine Ear Health and Hearing Checks

Round 1 draft recommendation

In Round 1, one draft recommendation about use of audiometry within routine Ear Health and Hearing Checks was presented, and one question was asked. These were:

Draft recommendation 4a: Screening audiometry is not recommended as part of Ear Health and Hearing Checks in primary health settings for children aged three years and younger.

Question 4b: Do you think screening audiometry should be recommended for Ear Health & Hearing Checks in primary health settings for children aged 4 to 5?

These questions were asked:

a) How strongly do you agree with the recommendation 'Screening audiometry is not recommended as part of Ear Health and Hearing Checks in primary health settings for children aged three years and younger'?
b) Any comments?
c) Do you think screening audiometry should be recommended for Ear Health & Hearing Checks in primary health settings for children aged 4 to 5?

d) Could you please explain why/why not?
e) Please comment on feasibility and implementation issues.

Key expert responses

Draft recommendation 4a:

56 respondents answered this question. The 80% consensus level was not reached. The chart below shows the full range of responses.

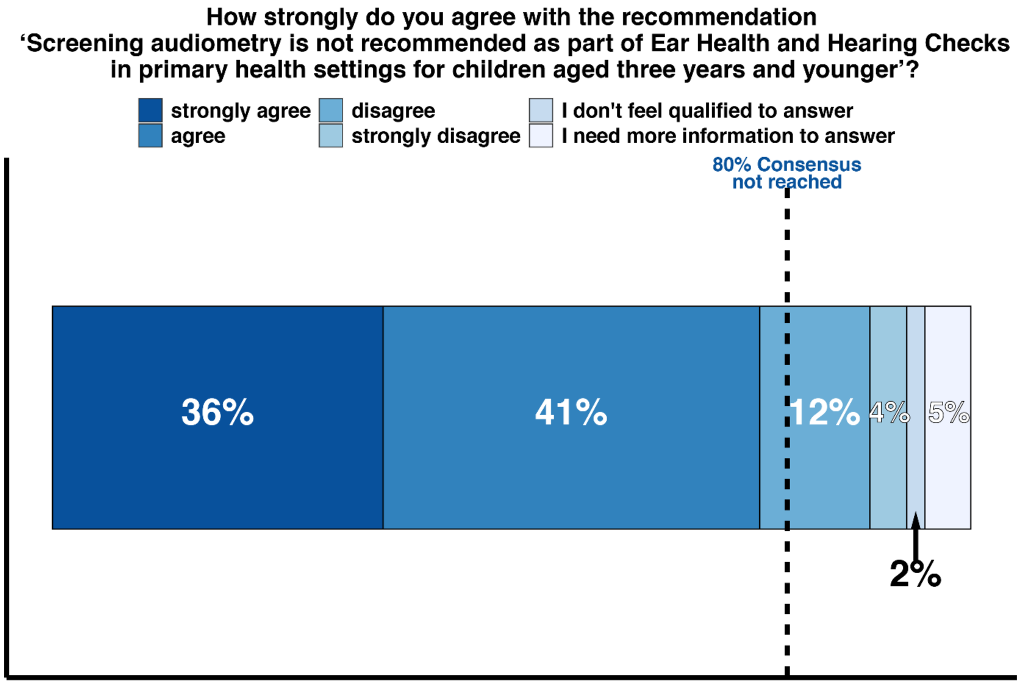

Question 4b

56 respondents answered this question. The 80% consensus level was not reached. The chart below shows the full range of responses.

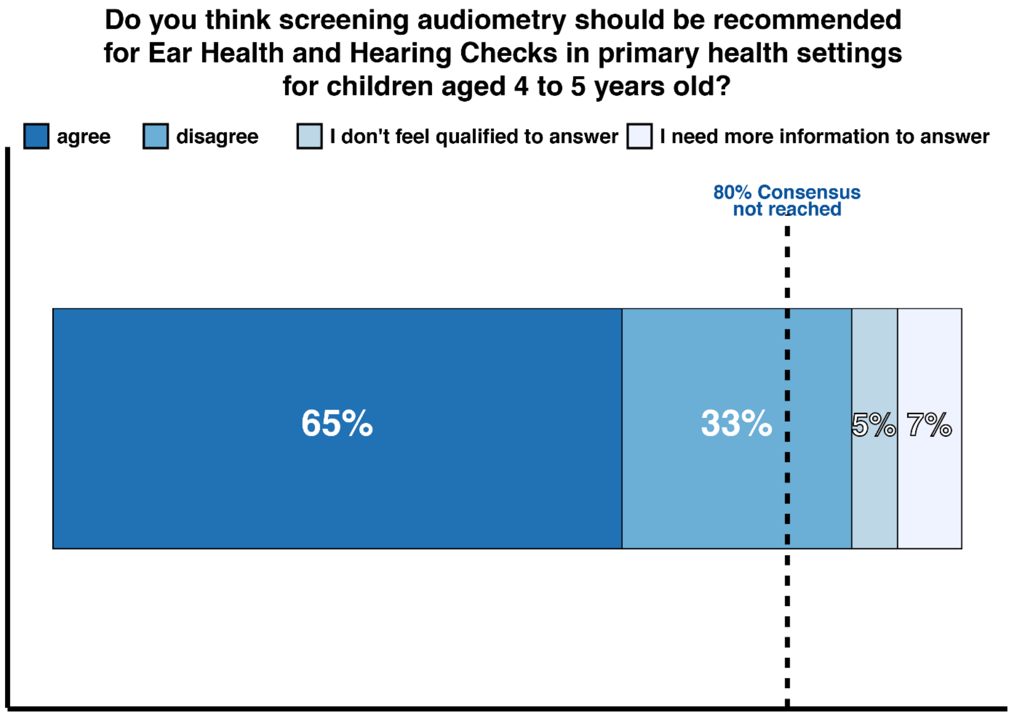

Themes of the comments

Analysis of comments for both draft recommendation (4a) and question (4b) indicated that many responses related to audiometry in the broader primary health setting (e.g. audiometry arranged for children with ear health or hearing concerns), rather than audiometry as part of routine Ear Health and Hearing Checks.

This consultation asks about the use of audiometry specifically in routine Ear Health and Hearing Checks, that is, for Aboriginal and Torres Strait Islander children aged under 6 years, who are not already being treated for ear health and/or hearing problems. This recommendation is not about the broader use of audiometry in primary health.

Themes of comments related to draft recommendation 4a*: Screening audiometry is not recommended as part of Ear Health and Hearing Checks in primary health settings for children aged three years and younger:*

- audiometry to screen hearing is necessary (for all ages)
- audiometry for under 3-year-olds requires training and skill maintenance
- audiometry for under 3-year-olds is impractical in the primary health context
- audiometry for under 3-year-olds should not be used in all checks
- indirect measures of hearing provide enough information, so audiometry for under 3-year-olds is not necessary.

Themes related to the question 'Do you think screening audiometry should be recommended for Ear Health & Hearing Checks in primary health settings for children aged 4 to 5?' in comments specifically about audiometry in routine Ear Health and Hearing Checks: audiometry should/could be used to screen before children start school

- audiometry can be challenging to conduct, especially for under 5-year-olds
- resources, equipment, and support are required
- there are rebate and funding concerns
- audiometry requires training and skill maintenance
- audiometry requires support from outside the primary health context
- time constraints in primary health settings affect feasibility
- there are risks to conducting and interpreting audiometry in primary health settings
- audiometry is not required in Ear Health and Hearing checks because other components can be used to identify children who need further assessment/inform next steps
- audiometry is not very feasible for (certain) primary health settings
- audiometry should not be used in all/routine Ear Health and Hearing Checks.

These above themes were also identified in comments relating to the role of audiometry with 4 to 5-year-olds in broader primary health settings. In addition, these themes were also identified:

Additional themes of comments relating to audiometry

- audiometry can have benefits in primary health settings
- audiometry is feasible to implement in primary health settings
- audiometry requires a clear pass/refer protocol
- audiometry should be used for all children over 3 years in primary health settings
- automated tools may be more feasible
- referral pathways to diagnostic audiometry are needed.

Round 2 revised draft recommendation

After reviewing results and feedback from Round 1, the following revised draft recommendation for audiometry is proposed:

Revised draft recommendation 5: *As part of routine Ear Health and Hearing Checks in primary health settings, audiometry is not recommended.*

Rationale for revised draft recommendation

Information from the rapid evidence review:

- The rapid evidence review found little direct evidence on the accuracy of pure tone audiometry in primary health settings to identify children with hearing loss. Variations in the accuracy of pure tone audiometry may be affected by the training and experience of screeners, the pass/refer thresholds selected, and the levels of environmental noise.
- Only two of the reviewed guidelines recommend including audiometry as part of routine ear health assessments in primary health settings.
- For older children (aged 4 to 5 years) it is possible for primary health practitioners to screen hearing using automated apps or manual screening audiometry.
- *The Otitis Media Guidelines for Aboriginal and Torres Strait Islander Children* recommend audiometry upon diagnosis of some forms of persistent Otitis Media, and when there are concerns about language, learning, behavioural or developmental problems. It is considered a next step when concerns are raised or found by primary health practitioners.
- Refer to Round 1 evidence summary in the audiometry section for further information.

Responses from the expert panel:

- Overall, expert panel responses indicated low levels of support for audiometry in Ear Health and Hearing Checks. Consensus was nearly reached for draft recommendation 4a: *Screening audiometry is not recommended as part of Ear Health and Hearing Checks in primary health settings for children aged three years and younger* (76.8%). In response to question 4b, just over half of respondents thought that audiometry should be included in Ear Health and Hearing Checks for children aged 4 to 5 years old (62.5%).
- Audiometry for 4-5-year-olds was regarded by key experts as more feasible in primary health settings than for 0–3-year-olds but many comments indicated that it would be challenging to include in routine Ear Health and Hearing Checks.
- Several respondents felt that the possibility of hearing loss, and the need for audiometry, can be indicated by other components of routine Ear Health and Hearing Checks, e.g. a combination of parent/carer concern, ear health history, OAEs if included, and results of listening and communication skills review.
- Some positive comments were made about audiometry (e.g., audiometry can be engaging for children and can provide hearing levels to assist direct referrals). However, these comments related to the use of audiometry more broadly in primary health settings, rather than in this specific routine Ear Health and Hearing Check.
- The themes identified in comments from expert panel members referring specifically to the inclusion of audiometry in routine Ear Health and Hearing Checks for 4-5-year-olds were cautious or concerned in nature. None of these expert panel members supported the routine inclusion of audiometry in all Ear Health and Hearing Checks.

Additional clarification/justification:

For 4–5-year-old children, aside from time for room and equipment set up, the time required to explain the process to/discuss results with parents and carers, teach children the audiometry task, and to obtain results is approximately 15 to 20 minutes.

Currently, audiologists and nurse audiometrists are the only workforces for whom audiometry for children aged 0-3 years is in scope of practice. Testing children of this age requires a specialised test process, purpose-designed equipment, and a quiet room that can be darkened, with few distractions. For children aged 3 years and younger, these considerations often make screening audiometry unfeasible within routine primary health Ear Health and Hearing Checks.

4.2 Whispered voice test

Round 1 question

In Round 1, one question was asked about the use of Whispered Voice test as part of routine Ear Health and Hearing Checks. This was:

Question 7: Do you agree with the project team's proposal that the Whispered Voice test is not appropriate for inclusion in Ear Health and Hearing Checks?

Summary of key expert responses

- Consensus was reached: 83.9% of respondents agreed that the Whispered Voice test is not appropriate for inclusion in Ear Health and Hearing Checks.

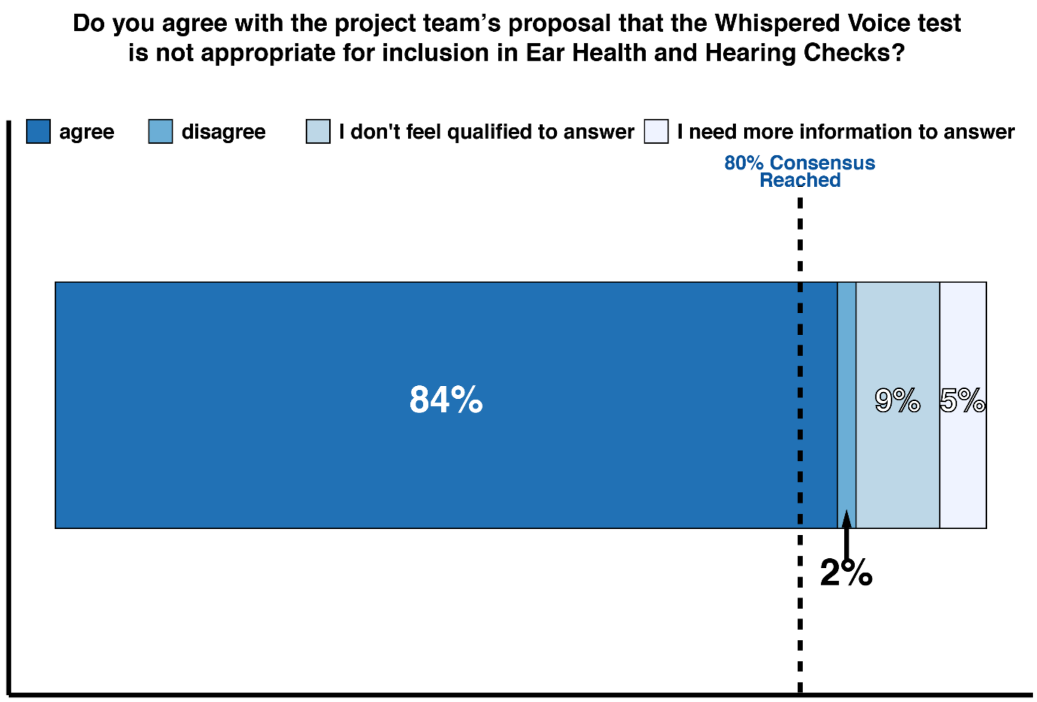

Themes of comments

- lack of reliability and evidence for Whispered voice test
- challenges in administering and interpreting
  - other tools can be used instead of Whispered voice test
  - Whispered voice test may be only option
  - Whispered voice test has benefits as a demonstration tool
  - Whispered voice test is simple

Outcome

The Whispered Voice Test is not recommended as a component of routine Ear Health and Hearing Checks. In round 2 we will not include further questions about the Whispered voice test.

4.3 Otoacoustic Emissions (OAEs) in routine Ear Health and Hearing Checks

Round 1 draft recommendation

In Round 1, the following draft recommendation was proposed:

Draft recommendation 7: Use otoacoustic emissions (OAE) testing to confirm or exclude normal or near-normal hearing

These questions were asked:

a) How strongly do you agree with this draft recommendation?

b) How feasible is it for this recommendation to be implemented in primary health settings?

Key expert responses

56 respondents answered this question. The 80% consensus level was not reached. The chart below shows the range of responses.

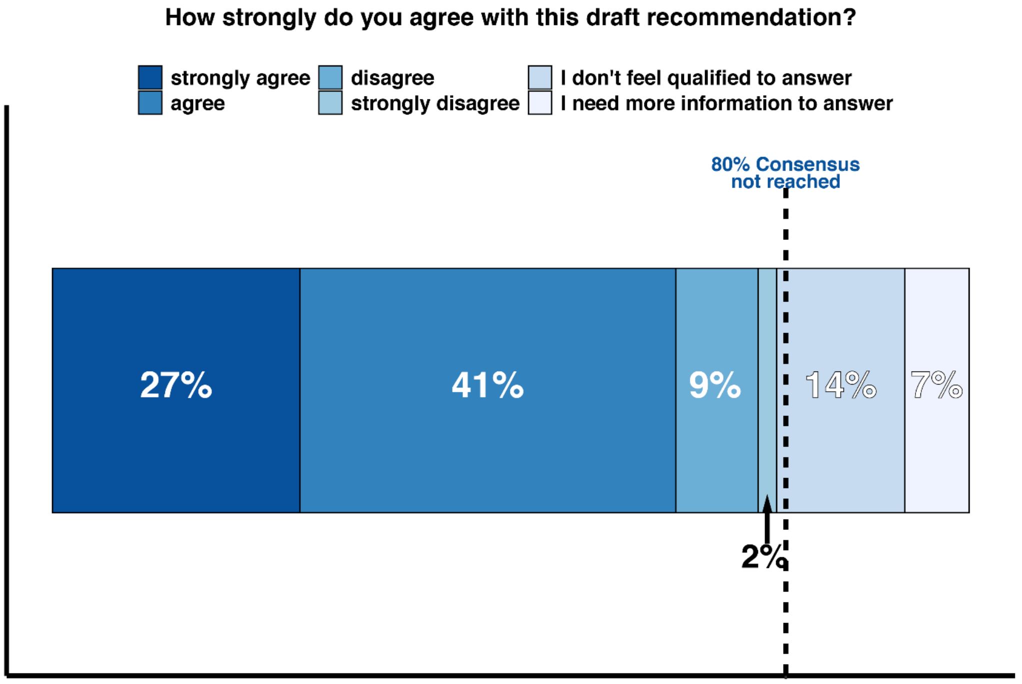

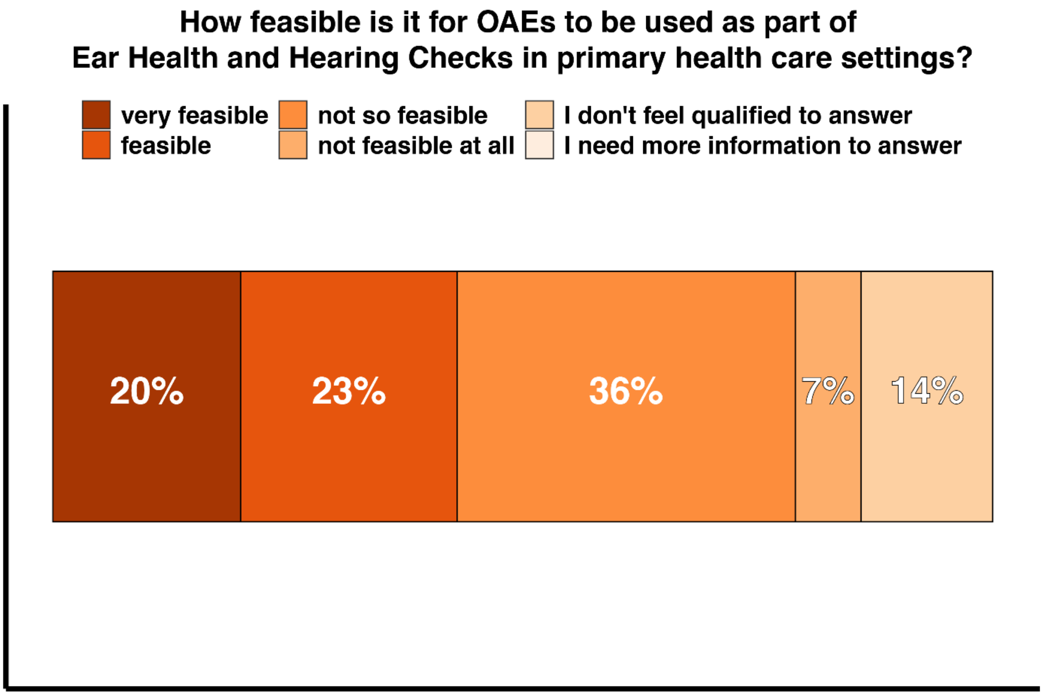

Themes of comments

Themes of comments positive about OAEs:

- OAEs are fast and easy
- OAEs are helpful for families and health workers
- OAEs have clinical merit for the population.

Themes of comments related to concerns about OAEs:

- concerns about adequate cost vs benefit ratio for OAEs
- concerns about buying, accessing, and maintaining equipment
- OAE recommendation needs guidance on age range/limits
- OAEs have risks (conducting and interpreting)
- OAEs are not feasible for all primary health settings
- OAEs are not helpful for families and health workers
- OAEs require training and skill maintenance
- rebates and funding concerns
- time constraint concerns.

Round 2 revised draft recommendation

After reviewing results and feedback from Round 1, the following revised draft conditional recommendation for OAEs is proposed, which makes OAE testing an optional component of routine Ear Health and Hearing Checks:

Revised draft recommendation 6: As part of routine Ear Health and Hearing Checks in primary health settings, Otoacoustic emissions (OAE) testing is suggested to confirm or exclude normal or near-normal hearing when:

- **equipment is available**
- **primary health practitioners have capability and are confident to use it**
- **there is a local preference for using OAE testing.**

Rationale for revised draft recommendation

Information from the rapid evidence review:

- The rapid evidence review found little direct evidence on the accuracy of OAEs in primary health settings to identify children with hearing loss and variations in accuracy may be affected by the training and experience of screeners.

Responses from the expert panel:

- Consensus was not reached on this recommendation. Only 68.7% of respondents agreed with the use of OAE testing to confirm or exclude normal or near-normal hearing in Ear Health and Hearing Checks and only 42.86% of respondents thought this recommendation was feasible.
- There was a range of views in the responses, from positive comments (particularly from health practitioners who already use OAEs) to comments with concerns about risks (in conducting and interpreting OAEs) and concerns about feasibility.
- Given the lack of consensus, the need to balance the different views and concerns, and the significant challenges of wide-scale implementation, the project team has proposed the revised draft conditional recommendation, making OAE testing an optional component of Ear Health and Hearing Checks.

Timing or scheduling of checks

Round 1 draft recommendation

In Round 1, the following draft recommendation relating to the timing of the checks was presented:

Draft recommendation 9: Following newborn hearing screening, Ear Health and Hearing Checks should be scheduled, at least, three monthly until 24 months of age (a combination of 'minimum' and 'full' checks), twice while aged two years, and annually at age three, four and five years.

Remember: These recommendations are for children who are not known to have OM. Any child with OM would/should be in a surveillance or active treatment clinical pathway.

These questions were asked:

a) How strongly do you agree with the recommendation for three-monthly checks in a child's first 24 months, alternating 'minimum' and 'full' checks? Insert check boxes
b) How feasible is it for this recommendation to be implemented in primary health settings?
c) Any comments?

d) How strongly do you agree with the recommended components of a 'minimum' check (i.e. review parent concern, recent history and assessment of ear drum/middle ear appearance and movement)?
e) Any comments, including on feasibility?

f) How strongly do you agree with the recommended components of a 'full' check (i.e. also includes review of listening and communication skills development using questionnaire/checklist, and OAEs when appropriate)?
g) Any comments, including on feasibility?

h) How strongly do you agree with the recommendation for two 'full' checks while aged two years (e.g. at 24 and 30 months)? Insert check boxes
i) How feasible is it for this recommendation to be implemented in primary health settings?
j) Any comments?

k) How strongly do you agree with the recommendation for annual 'full' checks at age three, four and five years? Insert check boxes
l) How feasible is it for this recommendation to be implemented in primary health settings?
m) Any comments?

Key expert responses

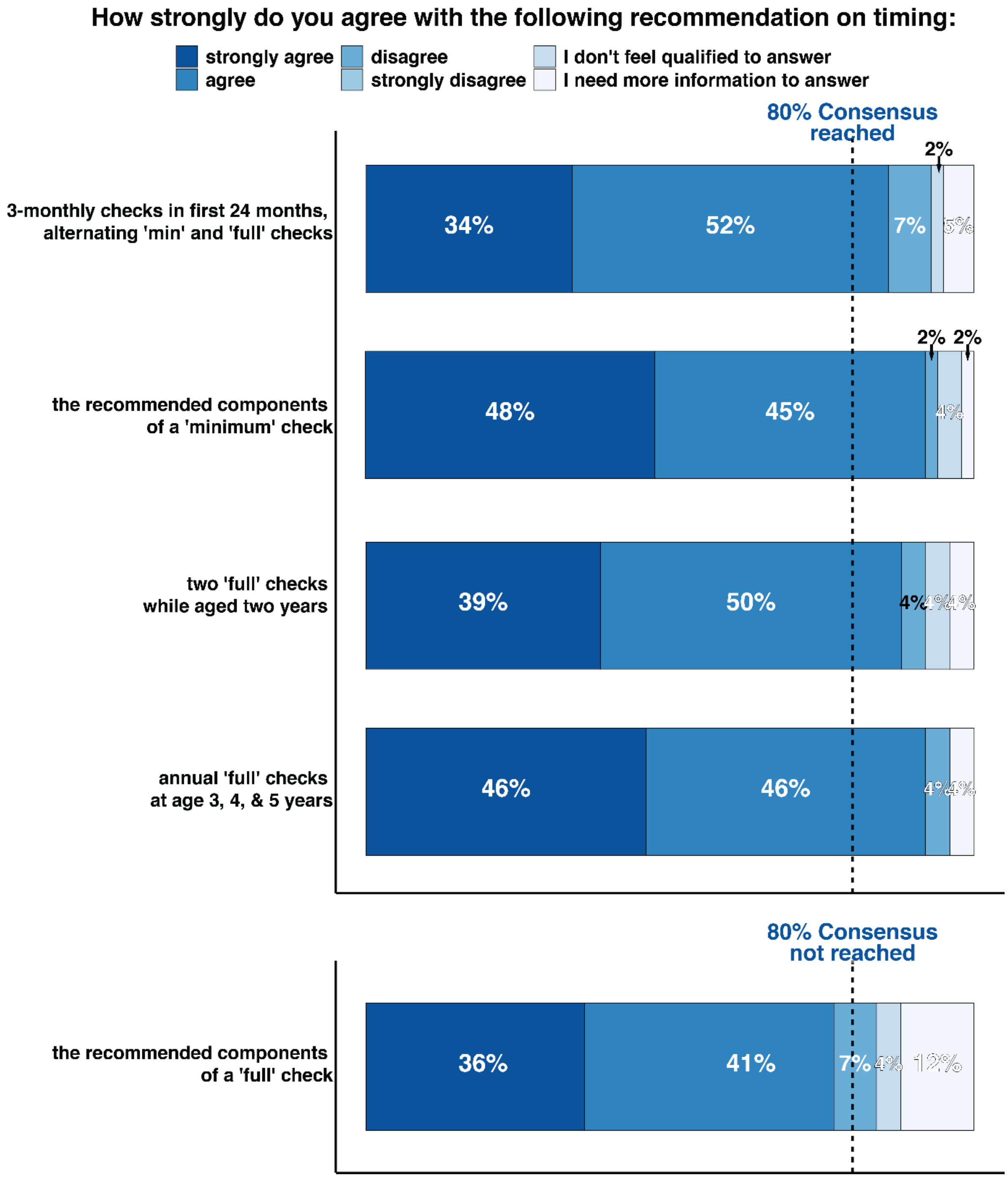

Key expert responses on feasibility of timing

3-monthly checks in the first 24 months

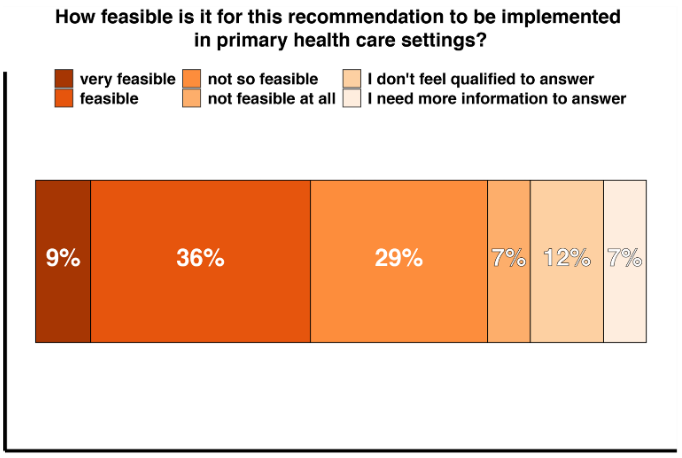

Two 'full' checks while aged 2 years

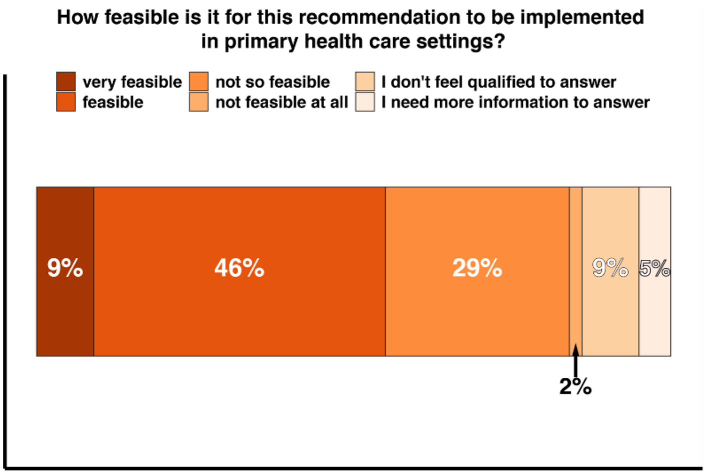

Annual 'full' checks at age 3, 4, & 5 years

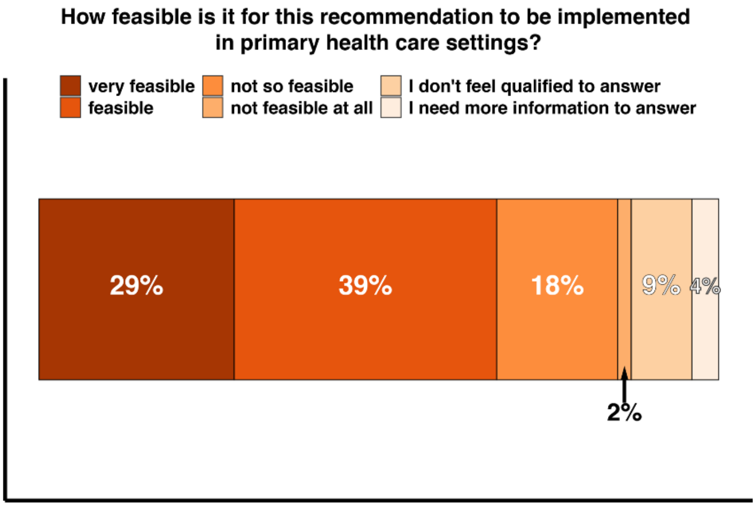

Themes of comments

Themes related to 'minimum' check components:

- minimum checks should only include asking caregivers about concerns and history
- minimum checks should include hearing sensitivity
- appearance and mobility are most important in a minimum check
- pneumatic otoscopy has risks

Themes related to 'full' check components:

- concerns about including OAEs in the full check
- OAEs could/should be in all checks (full and minimum)
- automated audiometry should be included in full checks

Themes related to both 'minimum' and 'full' checks:

- the checks (or components of them) have benefits for health promotion/education/awareness
- concerns about the challenges of checks for families (checks may be onerous; parents/carers less likely to engage unless there are concerns)
- concerns about feasibility of checks (related to: funding, referral pathways, time constraints, workforce)
- checks need to link to existing health checks

Themes relating to 'three-monthly checks in a child's first 24 months, alternating 'minimum' and 'full checks'':

- three-monthly checks are optimal/beneficial
- concerns about the feasibility and effectiveness of three-monthly checks
- checks need to be consistent

Themes relating to 'two 'full' checks while aged two years (e.g. at 24 and 30 months)':

- concerns about the challenges of checks for younger ages

Themes relating to 'annual 'full' checks at age three, four and five years':

- concerns about the timing of checks (number rather than specific times would be better, more frequent checks needed for younger children/those at greater risk)
- concerns about the components of 'full' checks (OAEs should only be for 3-year-olds who can't do play audiometry)

Comments related to linkage to the Ear Health and Hearing Checks in general:

- checks need to link with existing health checks
- concerns about feasibility of checks (related to equipment, funding, time constraints, space, workforce)
- later review/evaluation of checks needed (after implementation)
- systemic changes are needed (checks need to be integrated into health systems, reporting on ear health and hearing is needed [like vaccination rates])

Round 2 revised draft recommendation

After reviewing Round 1results and feedback, the following revised draft recommendations are proposed:

Revised draft recommendation 7: Following newborn hearing screening, Ear Health and Hearing Checks should be scheduled at least 6 monthly until the age of 4 and then one check at 5 years old.

Draft (Conditional) Recommendation 8: It is suggested that Ear Health and Hearing Checks be undertaken more frequently than 6 months:

- **in high-risk settings, and/or**
- **for children aged under 2 years, and/or**
- **when it is acceptable to families, and/or**
- **in response to parent/caregiver concerns.**

Rationale for new timing recommendation and conditional recommendation.

Information from the rapid evidence review:

- As outlined in the round 1 survey (see link here), little evidence exists on the optimal timing of Ear Health and Hearing Checks from other guidelines, where the timing of checks varies and may link with other schedules (e.g., for vaccination).

- Background evidence was presented in round 1 about the first two years of life as an intense period of language and communication development, which influenced the project team's decision to propose 3-monthly checks during this period. However, the team recognises the need to balance this with the feasibility of checks.

Responses from the expert panel:

- Consensus was reached on all parts of the original recommendation, except for the components of a full check (particularly concerns about the inclusion of OAEs in the full check, which has been revised).
- While some respondents felt that 3-monthly checks were optimal or beneficial, many respondents expressed concerns about the feasibility and effectiveness of three-monthly checks.
- Respondents also expressed concerns about the challenges of checks for families.
- In response to the expert panel's responses and comments, the project team revised the recommendation to be minimum 6-monthly Ear Health and Hearing Checks, and all Checks having the same components (since the components have been revised, as outlined above).
- The project team also included a conditional recommendation for more frequent checks in particular contexts.

Additional clarification/justification:

- The proposed routine Ear Health and Hearing Checks are for Aboriginal and Torres Strait Islander children who are not known to have OM or hearing problems, both to pick up any problems or to reassure families that there are no problems.
- An appropriate clinical response is required if any concerns about ear health or hearing are found during routine Ear Health and Hearing Checks.
- Any child with OM or a hearing problem should be in an active clinical pathway, addressed by relevant clinical Guidelines.
- A child who has been in an active clinical pathway (for OM and any associated hearing loss) and whose condition has been resolved should return to routine Ear Health and Hearing Checks.
- A minimum of 6-monthly routine Ear Health and Hearing checks is proposed so that more children who have ear and hearing problems will be identified and fewer children who have ear and hearing problems will be undiagnosed.
- The project team has included a conditional recommendation that additional Ear Health and Hearing checks be carried out: in high-risk settings, and/or for children aged under 2 years, and/or when it is acceptable to families, and/or in response to parent/carer concerns.

Overview of outcomes of Round 2 of the Ear Health and Hearing Check key expert consultation

Foreword

The routine Ear Health and Hearing Checks these recommendations refer to are intended to be undertaken by primary healthcare practitioners as part of standard care, whether parents/carers have expressed concern about their child's ear health or hearing, or not.

'Routine' in the context of these checks refers to both timing and components of Ear Health and Hearing Checks. The exception to this is otoacoustic emissions and video otoscopy, which are undertaken conditionally, or in certain circumstances.

As a reminder, this activity relates to the components and timing of routine Ear Health and Hearing Checks for:

- Aboriginal and Torres Strait Islander children
- aged under 6 years
- attending primary healthcare settings, and
- who are not already being treated for ear health and/or hearing problems because:
  - they are not known to have OM and/or hearing loss, or
  - they have been recognised as having OM and/or hearing loss but are not in active care.

It is critical that these recommendations provide primary health practitioners with sufficient information to decide whether a child needs further assessment, triaging for referral, or close monitoring.

Participants in the Round 2 consultation

51 key experts either fully (49) or partially completed the Round 2 consultation survey. Primary health was well represented: 50% of responses were completed by members of the key expert panel who work in this sector, just over half of whom (56%) work in the Aboriginal and Torres Strait Islander community-controlled sector.

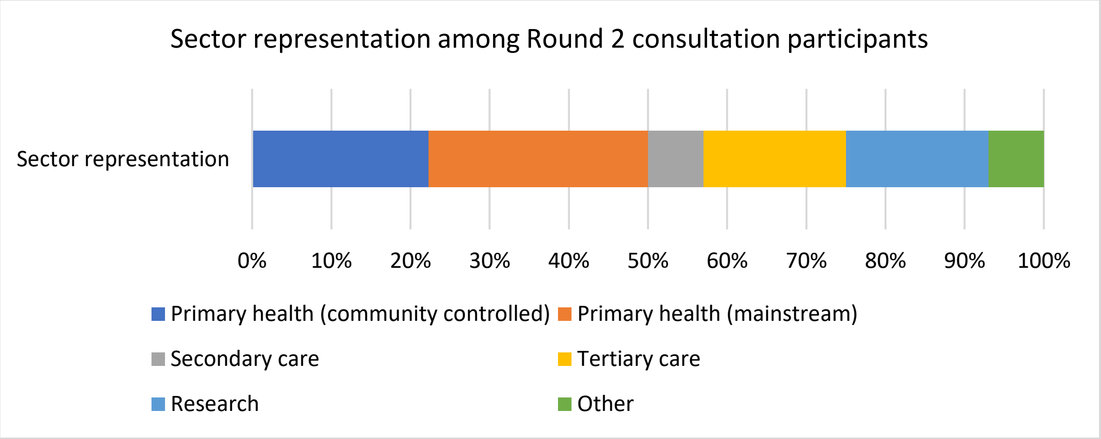

Participants were evenly distributed across remoteness areas.

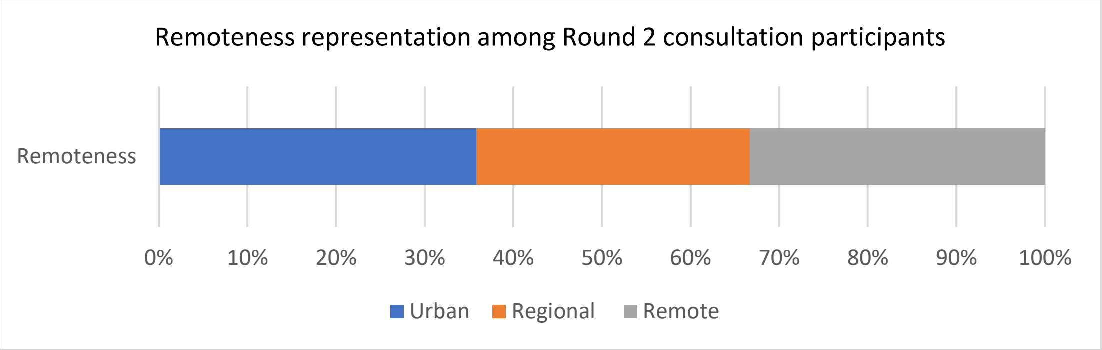

Proposed goals of routine Ear Health and Hearing Checks

Round 1 draft recommendation

In round 1, the following goals of routine Ear Health and Hearing Checks were proposed:

9. Identify children who have good ear health, hearing, and listening and communication development
10. Identify children who have an acute or persistent ear health condition
11. Identify children who may be experiencing hearing loss
12. Identify children whose listening and hearing-related communication development may be delayed
13. Identify children who need further ear health and hearing assessment
14. Provide an opportunity for parents/carers to talk about children's ear health and hearing
15. Build rapport between health practitioners and parents/carers
16. Build knowledge of ear health, hearing, listening and communication development among parents/carers.

Level of agreement with proposed goals

In Round 1, consensus agreement was reached for each of the goals: between **86** to **100%** agreement.

Domain 1: Parent and carer-reported history, concerns, signs, and symptoms

Draft recommendation 1:

As part of routine Ear Health and Hearing Checks in primary health care settings, ask parents/carers about: 1) their child's ear health (recent and longer term); 2) any concerns about their child's ear health, hearing, or communication.

Level of agreement with recommendation from Round 1

In Round 1, **96%** of respondents agreed with the draft recommendation: **consensus was reached.**

Responses to Round 2 question on feasibility

In Round 2, **92%** of respondents agreed the recommendation is feasible to implement in primary health settings

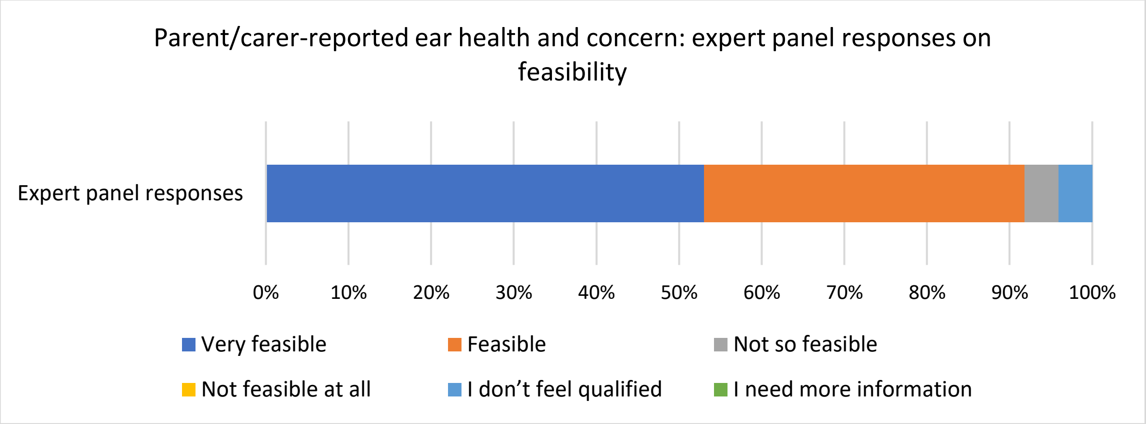

Themes from analysis of comments

- Community and family awareness needed
- Trusting relationships with primary health needed
- Caregiver concerns should always be acted upon
- Caregivers may find it challenging to respond to questions about concerns and history
- Training required
- Clarity around recommendation needed

Domain 2: Listening and communication skills

Draft recommendation 2

As part of routine Ear Health and Hearing Checks in primary health care settings, from the age of six months, review children's listening and communication skills development with parents/carers using appropriate questionnaires or checklists.

Level of agreement with recommendation from Round 1

In Round 1, 98% of respondents agreed with the draft recommendation: **consensus was reached.**

Responses to Round 2 question on feasibility

In Round 2, **88%** of respondents agreed the recommendation is feasible to implement in primary health settings.

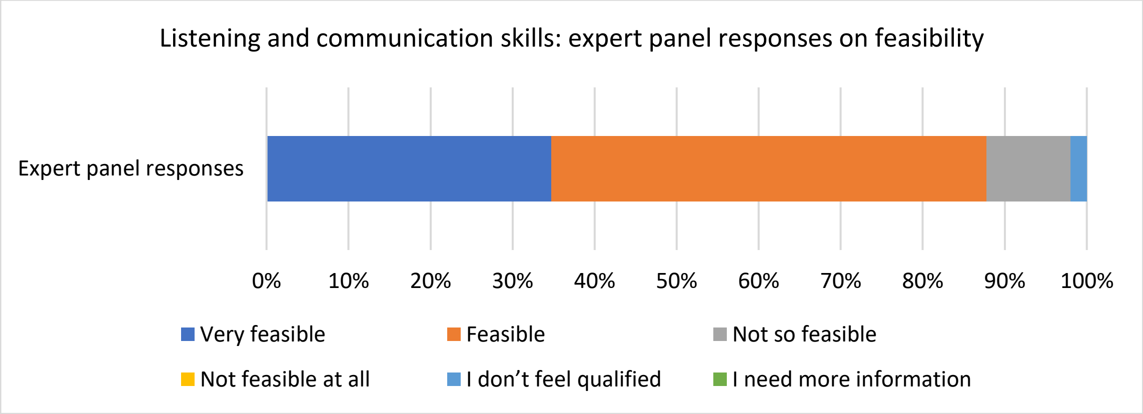

Themes from analysis of Round 2 comments on feasibility

- Trusting relationships with primary health needed
- Community and family awareness needed
- Questionnaires can be part of a conversation

- Questionnaires need to be co-developed
- Length of questionnaires need to be feasible for workload
- Questionnaires need to be culturally, linguistically, and developmentally appropriate
- Structured questionnaires improve communication between health workers and carers
- PLUM and HATS useful
- Electronic documentation (e.g., in My Health Record) required for questionnaires
- More trained staff needed
- Referral and support pathways needed
- Consistency in use of questionnaires preferred

Domain 3: Ear health

> ***Draft recommendation 3***
>
> As part of routine Ear Health and Hearing Checks in primary health care settings, examine the appearance of the ear canal and ear drum, and assess the movement of the ear drum and middle ear using either simple otoscopy plus tympanometry OR pneumatic otoscopy.

Level of agreement with recommendation from Round 1

In Round 1,

- **97%** of respondents agreed with examining the appearance of the ear drum
- **91%** agreed with examining the movement of the ear drum
- **93%** agreed with examining both movement and appearance of the eardrum as part of routine Ear Health and Hearing checks

Consensus was reached.

Responses to Round 2 question on feasibility

In Round 2, **82%** of respondents agreed the recommendation is feasible to implement in primary health settings.

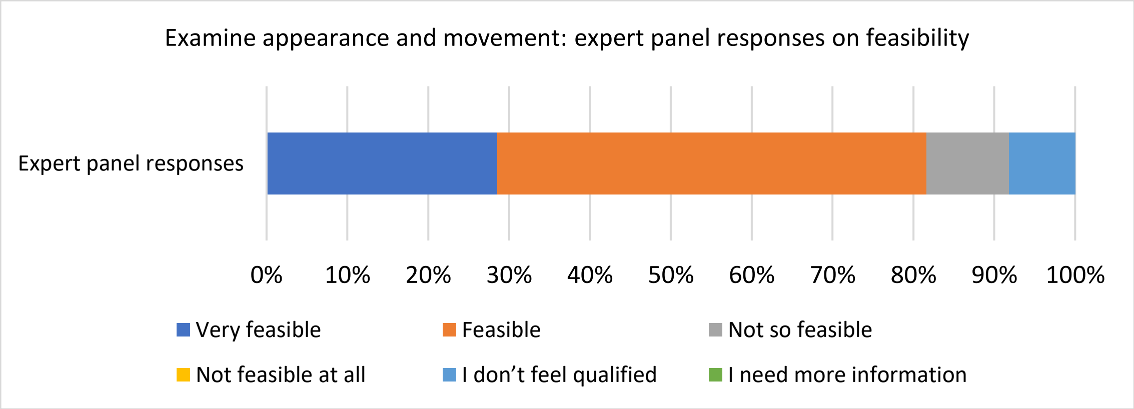

Themes from analysis of Round 2 comments on feasibility

- Community and family awareness needed
- Otoscopy and Tympanometry requires training and ongoing skill maintenance
- Equipment and maintenance access/costs/burden
- Pneumatic Otoscopy is inexpensive
- Tympanometry is preferable to pneumatic otoscopy (for diagnostic value)
- Tympanometry is preferable to pneumatic otoscopy (PO difficult to learn)
- Otoscopy and Tympanometry is challenging for children under 6 months
- Funding and rebates required

– The recommended Ear Health and Hearing Check is too much to attend to and document in one appointment

Draft (conditional) recommendation 4

As part of routine Ear Health and Hearing Checks in primary health settings, use of video otoscopy is suggested for health promotion purposes with parents/carers, and/or for sharing images with other health care practitioners.

Level of agreement with recommendation from Round 1

In Round 1, **96%** of respondents agreed with the draft recommendation: **consensus was reached.**

Responses to Round 2 question on feasibility

In Round 2, **71%** of respondents agreed the recommendation is feasible to implement in primary health settings.

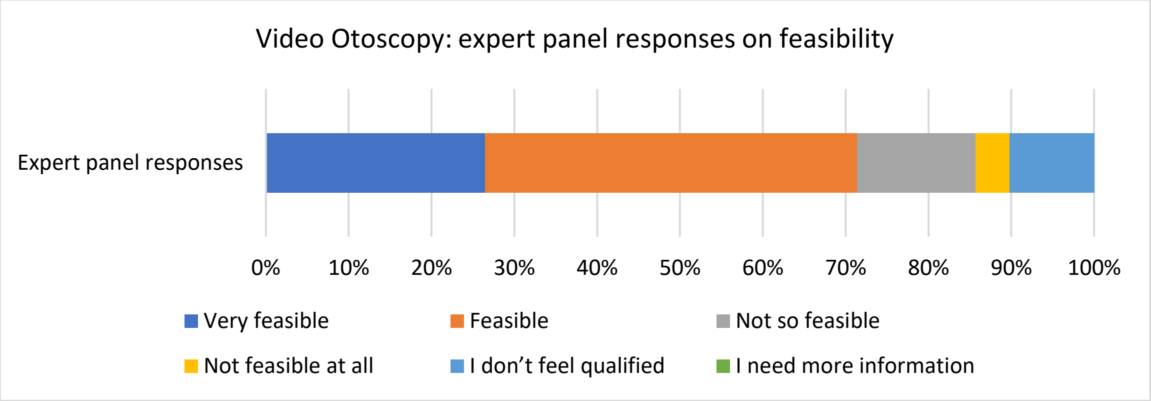

Themes from analysis of Round 2 comments on feasibility

- Video Otoscopy requires ongoing training and skill maintenance
- Equipment and maintenance access/costs/burden
- Video Otoscopy requires clinical software and secure transmission of images
- Video Otoscopy is not feasible for home visiting
- Video Otoscopy is feasible (easy to use, affordable)
- Video Otoscopy is beneficial (clinically, for health education and awareness, for tracking over time, when ENT not available)

Domain 4: Hearing sensitivity

Draft (conditional) recommendation 5

As part of routine Ear Health and Hearing Checks in primary health care settings, otoacoustic emissions (OAE) testing is suggested to confirm or exclude normal or near-normal hearing when:

- equipment is available
- primary health practitioners have capability and are confident to use it
- there is a local preference for using OAE testing.

Level of agreement with recommendation from Round 2

In Round 2, **84.3%** of respondents agreed with the OAE recommendation. **Consensus was reached.**

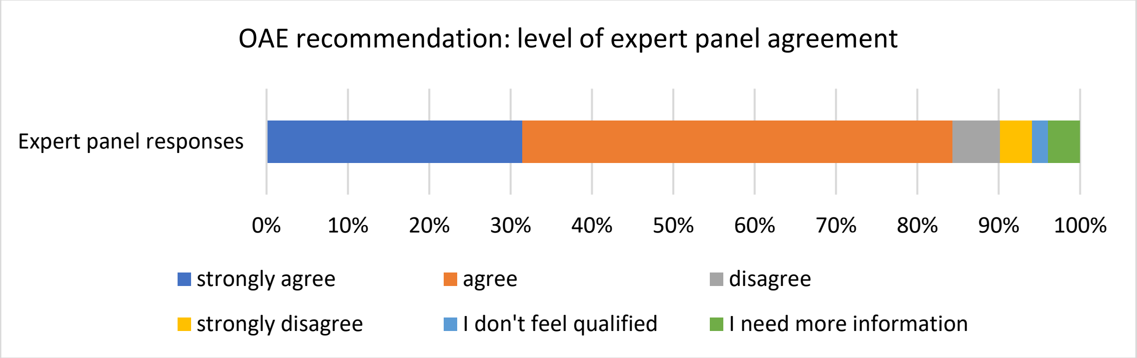

Responses to Round 2 question on feasibility

In Round 2, **75%** of respondents agreed the recommendation is feasible to implement in primary health settings.

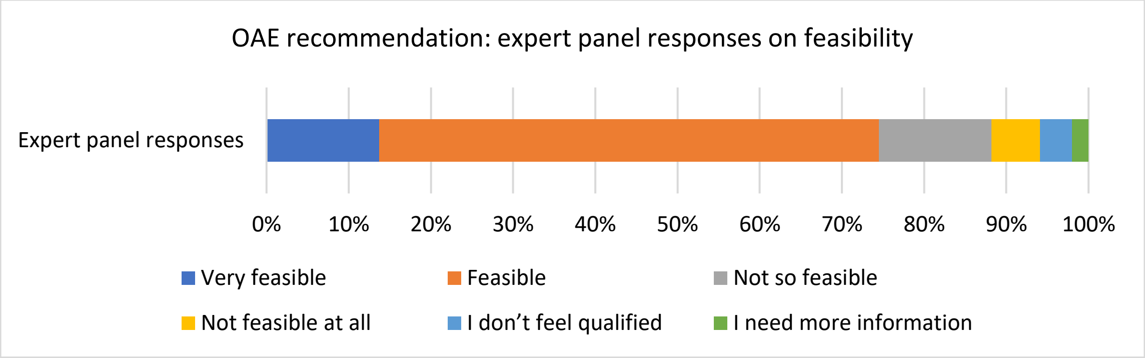

Themes from analysis of Round 2 comments on feasibility

- OAEs have clinical merit
- OAEs require training and skill maintenance
- OAEs require clear specific protocols
- OAE equipment is costly
- OAEs are feasible
- OAEs not feasible for all primary health settings
- OAEs can be difficult
- OAEs have risks (conducting and interpreting)
- OAEs require a systems approach to implement
- Accessible referral pathways needed
- Clarity on recommendation is required
- Audiometry more important than OAEs
- Rebates and funding concerns

Timing of Ear Health and Hearing Checks

Draft recommendation 6

Following newborn hearing screening, Ear Health and Hearing Checks are recommended at least 6 monthly until the age of 4 years, and then one check at 5 years old.

Level of agreement with recommendation from Round 2

In Round 2, **87.8%** of respondents agreed with the Timing recommendation. **Consensus was reached.**

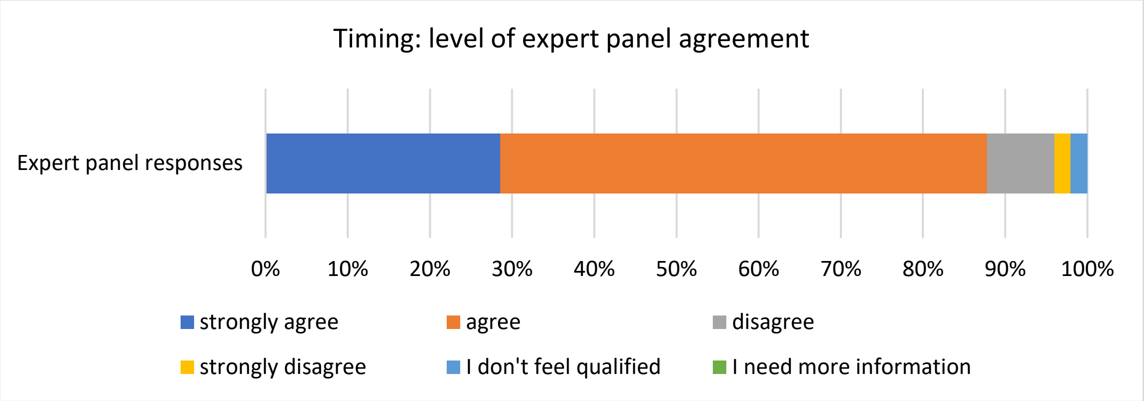

Responses to Round 2 question on feasibility

In Round 2, **67%** of respondents agreed the recommendation is feasible to implement in primary health settings.

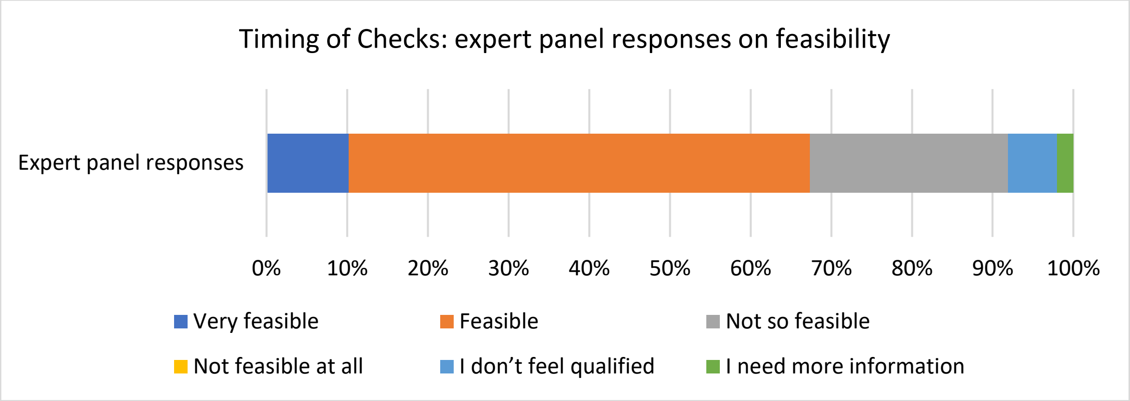

Themes from analysis of Round 2 comments on feasibility

- Trusting relationships with primary health needed
- Public health campaign needed
- Community and family awareness needed
- Concerns about the feasibility of checks (workforce, resources, funding)
- Opportunistic checks should be encouraged
- 3-monthly/more frequent checks are optimal/beneficial
- 6-monthly checks are feasible (given the recommended components)
- 12-monthly checks are optimal
- Checks are cost-effective considering long-term benefit
- Concerns about the challenges of checks for families
- Clarity needed in timing recommendation
- Timely access to pathways required

Draft (conditional) recommendation 7

It is suggested that Ear Health and Hearing Checks be undertaken more frequently than 6 months:

- in high-risk settings, and/or
- for children aged under two years, and/or
- when it is acceptable to families, and/or
- in response to parent/carer concerns.

Level of agreement with recommendation from Round 2

In Round 2, 88% of respondents agreed with the conditional Timing recommendation. **Consensus was reached.**

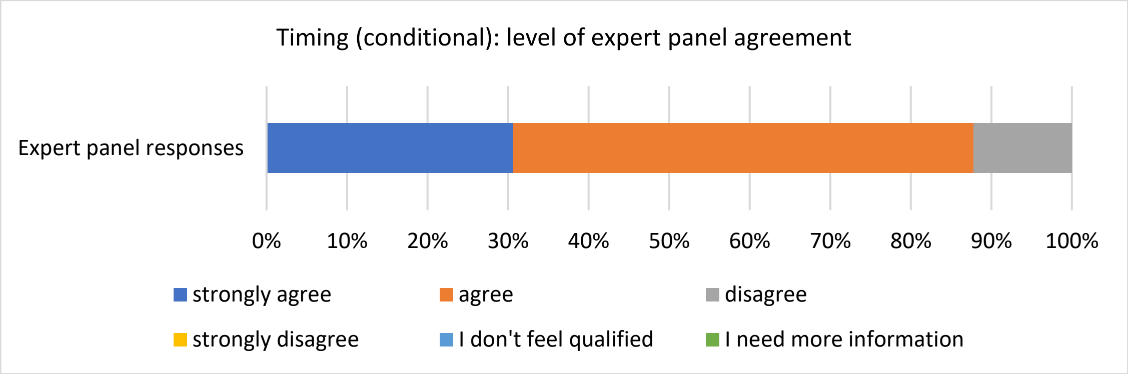

Responses to Round 2 question on feasibility

In Round 2, **64%** of respondents agreed the recommendation is feasible to implement in primary health settings.

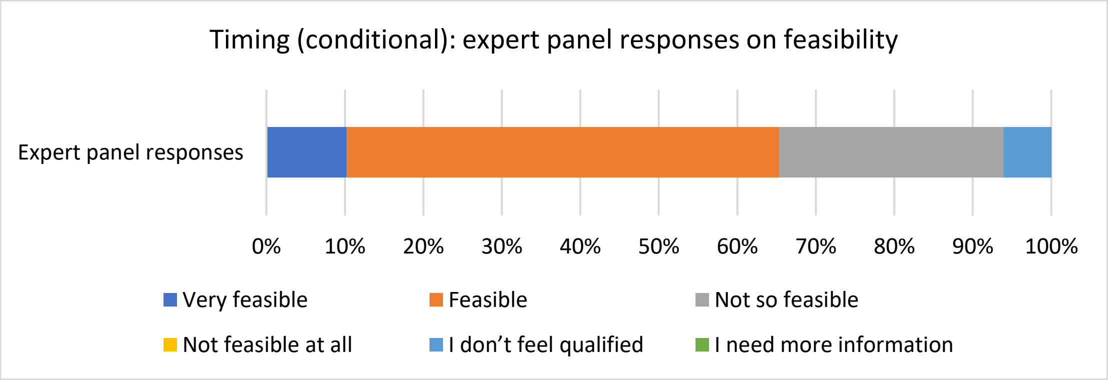

Themes from analysis of Round 2 comments on feasibility

- Community and family awareness needed
- Strengths-based approach needed
- Concerns about the feasibility of checks (resources, workforce, funding)
- Clarity needed in timing recommendation
- Concerns about the challenges of checks for families
- 3-monthly/more frequent checks are optimal/beneficial
- More frequent checks could be challenging
- Opportunistic checks should be encouraged
- Checks are cost-effective considering long-term benefit
- Checks need to link to existing health checks
- Timely access to pathways required
- Audiometry needed when children are high risk
- PLUM and HATS checklists useful for children at high risk

Audiometry

Draft recommendation

As part of routine Ear Health and Hearing Checks in primary health care settings, audiometry is not recommended.

Level of agreement with recommendation from Round 2

In Round 2, **71%** of respondents agreed with the Audiometry recommendation. **Consensus agreement was not reached.**

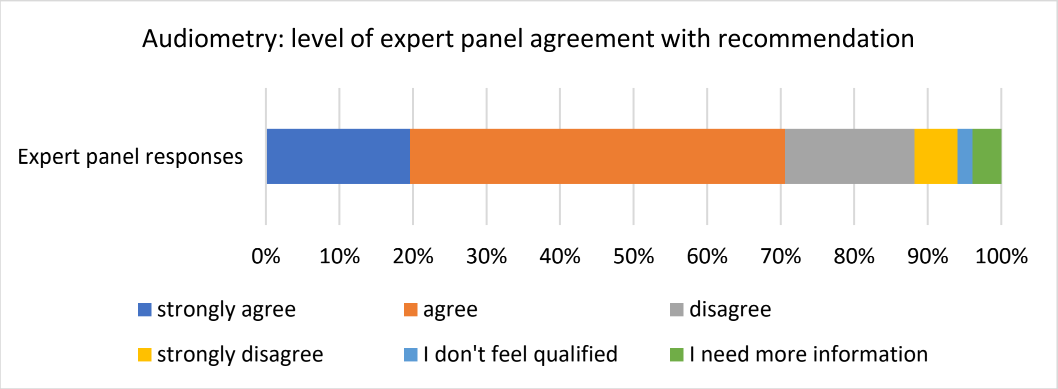

Themes from analysis of Round 2 comments on feasibility

Themes of comments of those who agreed with the recommendation

– Accessible referral pathways needed
– Audiometry is not an indication of cumulative hearing loss
– Audiometry is only needed when there are indications of OM or hearing impairment
– Audiometry requires training and skill maintenance
– Audiometry is feasible for 4-5-year-olds with the right training and equipment
– Audiometry should not be done for under 3-year-olds
– Clarity needed on recommendation
– Clinical governance is required for audiometry
– Concerns about feasibility (cost, time)
– School-aged screening audiometry is too late

Themes of comments of those who disagreed with the recommendation

– Accessible referral pathways needed
– Audiometry provides valuable information (but is age-related)
– Audiometry only needed when there are indications of OM or hearing impairment
– Audiometry requires training and skill maintenance
– Clarity needed on recommendation
– Concerns about feasibility (cost)
– Screening audiometry is easy
– Screening audiometry should be done for 4+ year-olds
– Screening audiometry should not be done for under 3s

Search strategies

Database: EBM Reviews - Cochrane Database of Systematic Reviews <2005 to July 14, 2021>

Search Strategy:

----

1 infant*.tw.

2 child*.tw.

3 (preschool* or pre-school*).tw.

4 nursery.tw.

5 p?ediatric.tw.

6 school student*.tw.

7 young person*.tw.

8 "0 to 6 years".tw.

9 "0-6 years".tw.

10 1 or 2 or 3 or 4 or 5 or 6 or 7 or 8 or 9

11 Middle ear disease*.tw.

12 Middle ear inflammation.tw.

13 Middle ear infection.tw.

14 Middle ear effusion.tw.

15 Glue ear.tw.

16 Otitis media*.tw.

17 (OM or OME).tw.

18 Acute otitis media*.tw.

19 (AOM or AOMwoP or AOMwiP).tw.

20 Chronic otitis media.tw.

21 Chronic suppurative otitis media.tw.

22 CSOM.tw.

23 Persistent otitis media*.tw.

24 POME.tw.

25 Serous otitis media.tw.

26 Secretory otitis media.tw.

27 hearing.tw.

28 Ear health.tw.

29 Hearing difficult*.tw.

30 ear discomfort.tw.

31 Listening difficult*.tw.

32 Hearing loss.tw.

33 HL.tw.

34 Conductive Hearing Loss.tw.

35 CHL.tw.

36 Hearing impair*.tw.

37 Hearing problem*.tw.

38 hard of hearing.tw.

39 Otoscop*.tw.

40 Pneumatic otoscop*.tw.

41 tympanometr*.tw.

42 acoustic reflectometr*.tw.

43 acoustic reflex.tw.

44 Otoacoustic emission*.tw.

45 audiometry.tw.

46 Pure tone audiometry.tw.

47 Pure tone screen*.tw.

48 Visual reinforcement Orientation Audiometry.tw.

49 VROA.tw.

50 Play audiometry.tw.

51 optical coherence tomography.tw.

52 OCT.tw.

53 Hearing screen*.tw.

54 Hearing assess*.tw.

55 hearing test*.tw.

56 hearing check*.tw.

57 ear check*.tw.

58 ear health check*.tw.

59 hearing surveillance.tw.

60 11 or 12 or 13 or 14 or 15 or 16 or 17 or 18 or 19 or 20 or 21 or 22 or 23 or 24 or 25 or 26 or 27 or 28 or 29 or 30 or 31 or 32 or 33 or 34 or 35 or 36 or 37 or 38 or 39 or 40 or 41 or 42 or 43 or 44 or 45 or 46 or 47 or 48 or 49 or 50 or 51 or 52 or 53 or 54 or 55 or 56 or 57 or 58 or 59

61 10 and 60

62 ("primary health care" or "primary healthcare").tw.

63 primary care.tw.

64 general practice*.tw.

65 family medicine.tw.

66 (mother and child health clinic*).tw.

67 (mother and child health center*).tw.

68 (mother and child health centre*).tw.

69 telemedicine.tw.

70 telehealth.tw.

71 (ehealth or e-health).tw.

72 (mhealth or m-health).tw.

73 mobile health.tw.

74 smartphone.tw.

75 aboriginal community controlled health*.tw.

76 (ACCHO or ACCHOs or ACCH or ACCHs).tw.

77 aboriginal medical service*.tw.

78 (AMS or AMSs).tw.

79 62 or 63 or 64 or 65 or 66 or 67 or 68 or 69 or 70 or 71 or 72 or 73 or 74 or 75 or 76 or 77 or 78

80 61 and 79

81 limit 80 to last 21 years

****

Scopus Advanced search

TITLE-ABS-KEY (infant* OR child* OR preschool* OR pre-school* OR nursery OR p#ediatric OR "school student*" OR "young person*" OR {0 to 6 years} OR {0-6 years}) AND TITLE-ABS-KEY ("Middle ear disease" OR "Middle ear diseases" OR "Middle ear inflammation" OR "Middle ear infection" OR "Middle ear effusion" OR "Glue ear" OR "Otitis media*" OR {OM} OR {OME} OR "Acute otitis media*" OR {AOM} OR {AOMwoP} OR {AOMwiP} OR "Chronic otitis media" OR "Chronic suppurative otitis media" OR {CSOM} OR "Persistent otitis media*" OR {POME} OR "Serous otitis media" OR "Secretory otitis media" OR hearing OR "Ear health" OR "Hearing difficult*" OR "ear discomfort" OR "Listening difficult*" OR "Hearing loss" OR {HL} OR "Conductive Hearing Loss" OR {CHL} OR "Hearing impair*" OR "Hearing problem*" OR "hard of hearing" OR otoscop* OR "Pneumatic otoscop*" OR tympanometr* OR "acoustic reflectometr*" OR "acoustic reflex" OR "Otoacoustic emission*" OR audiometry OR "Pure tone audiometry" OR "Pure tone screen*" OR "Visual reinforcement Orientation Audiometry" OR {VROA} OR "Play audiometry" OR "optical coherence tomography" OR {OCT} OR "Hearing screen*" OR "Hearing assess*" OR "hearing test*" OR "hearing check*" OR "ear check*" OR "ear health check*" OR "hearing surveillance") AND TITLE-ABS-KEY ("primary health care" OR "primary healthcare" OR "primary care" OR "general practice*" OR "family medicine" OR "mother and child health clinic*" OR "mother and child health center*" OR "mother and child health centre*" OR telemedicine OR telehealth OR ehealth OR e-health OR mhealth OR m-health OR "mobile health" OR smartphone OR "aboriginal community controlled health*" OR {ACCHO} OR {ACCHOs} OR {ACCH} OR {ACCHs} OR "aboriginal medical service*" OR {AMS} OR {AMSs}) AND (LIMIT-TO (PUBYEAR , 2021) OR LIMIT-TO (PUBYEAR , 2020) OR LIMIT-TO (PUBYEAR , 2019) OR LIMIT-TO (PUBYEAR , 2018) OR LIMIT-TO (PUBYEAR , 2017) OR LIMIT-TO (PUBYEAR , 2016) OR LIMIT-TO (PUBYEAR , 2015) OR LIMIT-TO (PUBYEAR , 2014) OR LIMIT-TO (PUBYEAR , 2013) OR LIMIT-TO (PUBYEAR , 2012) OR LIMIT-TO (PUBYEAR , 2011) OR LIMIT-TO (PUBYEAR , 2010) OR LIMIT-TO (PUBYEAR , 2009) OR LIMIT-TO (PUBYEAR , 2006) OR LIMIT-TO (PUBYEAR , 2005) OR LIMIT-TO (PUBYEAR , 2004) OR LIMIT-TO (PUBYEAR , 2003) OR LIMIT-TO (PUBYEAR , 2002) OR LIMIT-TO (PUBYEAR , 2001) OR LIMIT-TO (PUBYEAR , 2000))

Database: Ovid MEDLINE(R) ALL <1946 to July 09, 2021>

Search Strategy:

----

1 Infant/

2 infant*.tw.

3 Child, Preschool/

4 child*.tw.

5 (preschool* or pre-school*).tw.

6 nursery.tw.

7 p?ediatric.tw.

8 school student*.tw.

9 young person*.tw.

10 "0 to 6 years".tw.

11 "0-6 years".tw.

12 1 or 2 or 3 or 4 or 5 or 6 or 7 or 8 or 9 or 10 or 11

13 exp Otitis Media/

14 Middle ear disease*.tw.

15 Middle ear inflammation.tw.

16 Middle ear infection.tw.

17 Middle ear effusion.tw.

18 Glue ear.tw.

19 Otitis media*.tw.

20 (OM or OME).tw.

21 Acute otitis media*.tw.

22 (AOM or AOMwoP or AOMwiP).tw.

23 Chronic otitis media.tw.

24 Chronic suppurative otitis media.tw.

25 CSOM.tw.

26 Persistent otitis media*.tw.

27 POME.tw.

28 Serous otitis media.tw.

29 Secretory otitis media.tw.

30 Hearing/

31 hearing.tw.

32 Ear health.tw.

33 Hearing difficult*.tw.

34 ear discomfort.tw.

35 Listening difficult*.tw.

36 Hearing Loss/

37 Hearing loss.tw.

38 HL.tw.

39 Hearing Loss, Conductive/

40 Conductive Hearing Loss.tw.

41 CHL.tw.

42 Hearing impair*.tw.

43 Hearing problem*.tw.

44 hard of hearing.tw.

45 Otoscopy/

46 Diagnostic Techniques, Otological/

47 Otoscop*.tw.

48 Pneumatic otoscop*.tw.

49 tympanometr*.tw.

50 acoustic reflectometr*.tw.

51 acoustic reflex.tw.

52 Otoacoustic emission*.tw.

53 exp Hearing Tests/

54 audiometry.tw.

55 Pure tone audiometry.tw.

56 Pure tone screen*.tw.

57 Visual reinforcement Orientation Audiometry.tw.

58 VROA.tw.

59 Play audiometry.tw.

60 Tomography, Optical Coherence/

61 optical coherence tomography.tw.

62 OCT.tw.

63 Hearing screen*.tw.

64 Hearing assess*.tw.

65 hearing test*.tw.

66 hearing check*.tw.

67 ear check*.tw.

68 ear health check*.tw.

69 hearing surveillance.tw.

70 13 or 14 or 15 or 16 or 17 or 18 or 19 or 20 or 21 or 22 or 23 or 24 or 25 or 26 or 27 or 28 or 29 or 30 or 31 or 32 or 33 or 34 or 35 or 36 or 37 or 38 or 39 or 40 or 41 or 42 or 43 or 44 or 45 or 46 or 47 or 48 or 49 or

50 or 51 or 52 or 53 or 54 or 55 or 56 or 57 or 58 or 59 or 60 or 61 or 62 or 63 or 64 or 65 or 66 or 67 or 68 or 69

71 12 and 70

72 Primary Health Care/

73 ("primary health care" or "primary healthcare").tw.

74 primary care.tw.

75 exp General Practice/

76 general practice*.tw.

77 family medicine.tw.

78 (mother and child health clinic*).tw.

79 (mother and child health center*).tw.

80 (mother and child health centre*).tw.

81 Maternal-Child Health Centers/

82 Telemedicine/

83 telemedicine.tw.

84 telehealth.tw.

85 (ehealth or e-health).tw.

86 (mhealth or m-health).tw.

87 mobile health.tw.

88 smartphone.tw.

89 Health Services, Indigenous/

90 aboriginal community controlled health*.tw.

91 (ACCHO or ACCHOs or ACCH or ACCHs).tw.

92 aboriginal medical service*.tw.

93 (AMS or AMSs).tw.

94 72 or 73 or 74 or 75 or 76 or 77 or 78 or 79 or 80 or 81 or 82 or 83 or 84 or 85 or 86 or 87 or 88 or 89 or 90 or 91 or 92 or 93

95 71 and 94

96 limit 95 to last 21 years

****

Informit Advanced search

Health related Indexing databases selected for search: APAIS-Health, Aboriginal and Torres Strait Islander Health Bibliography, Australian Policy Observatory Database, Australasian Medical Index, APAFT, Health-Society, Indigenous Australia, Indigenous Studies Bibliography, Rural and Remote Health Database

Advanced search string

[[Title: child* OR Title: infant*] AND [Title: 'middle ear' OR Title: hearing*]] OR [[Abstract: child* OR Abstract: infant*] AND [Abstract: 'middle ear' OR Abstract: hearing*]] AND Publication Date: (01/01/2000 TO 12/31/2020) AND Resource Type: Journal

Full text databases selected for search: Health Collection

Advanced search string

[[Title: child* OR Title: infant*] AND [Title: 'middle ear' OR Title: hearing*]] OR [[Abstract: child* OR Abstract: infant*] AND [Abstract: 'middle ear' OR Abstract: hearing*]] AND Publication Date: (01/01/2000 TO 12/31/2020) AND Resource Type: Journal

Limits selected: publication date 2000-2021, Resource type: Journal

Database: Embase Classic <1947 to 1973>, Embase <1974 to 2021 July 09>

Search Strategy:

----

1 infant/

2 infant*.tw.

3 toddler/

4 preschool child/

5 child*.tw.

6 (preschool* or pre-school*).tw.

7 nursery.tw.

8 p?ediatric.tw.

9 school student*.tw.

10 young person*.tw.

11 "0 to 6 years".tw.

12 "0-6 years".tw.

13 1 or 2 or 3 or 4 or 5 or 6 or 7 or 8 or 9 or 10 or 11 or 12

14 exp otitis media/

15 Middle ear disease*.tw.

16 Middle ear inflammation.tw.

17 Middle ear infection.tw.

18 Middle ear effusion.tw.

19 Glue ear.tw.

20 Otitis media*.tw.

21 (OM or OME).tw.

22 Acute otitis media*.tw.

23 (AOM or AOMwoP or AOMwiP).tw.

24 Chronic otitis media.tw.

25 Chronic suppurative otitis media.tw.

26 CSOM.tw.

27 Persistent otitis media*.tw.

28 POME.tw.

29 Serous otitis media.tw.

30 Secretory otitis media.tw.

31 hearing/

32 hearing.tw.

33 Ear health.tw.

34 Hearing difficult*.tw.

35 ear discomfort.tw.

36 Listening difficult*.tw.

37 hearing impairment/

38 Hearing loss.tw.

39 HL.tw.

40 exp conduction deafness/

41 Conductive Hearing Loss.tw.

42 CHL.tw.

43 Hearing impair*.tw.

44 Hearing problem*.tw.

45 hard of hearing.tw.

46 otoscopy/

47 auditory system examination/

48 Otoscop*.tw.

49 Pneumatic otoscop*.tw.

50 tympanometr*.tw.

51 tympanometry/

52 reflectometry/

53 acoustic reflectometr*.tw.

54 acoustic reflex/

55 acoustic reflex.tw.

56 evoked otoacoustic emission/

57 Otoacoustic emission*.tw.

58 exp hearing test/

59 audiometry.tw.

60 Pure tone audiometry.tw.

61 Pure tone screen*.tw.

62 Visual reinforcement Orientation Audiometry.tw.

63 VROA.tw.

64 Play audiometry.tw.

65 optical coherence tomography/

66 optical coherence tomography.tw.

67 OCT.tw.

68 Hearing screen*.tw.

69 Hearing assess*.tw.

70 hearing test*.tw.

71 hearing check*.tw.

72 ear check*.tw.

73 ear health check*.tw.

74 hearing surveillance.tw.

75 14 or 15 or 16 or 17 or 18 or 19 or 20 or 21 or 22 or 23 or 24 or 25 or 26 or 27 or 28 or 29 or 30 or 31 or 32 or 33 or 34 or 35 or 36 or 37 or 38 or 39 or 40 or 41 or 42 or 43 or 44 or 45 or 46 or 47 or 48 or 49 or 50 or 51 or 52 or 53 or 54 or 55 or 56 or 57 or 58 or 59 or 60 or 61 or 62 or 63 or 64 or 65 or 66 or 67 or 68 or 69 or 70 or 71 or 72 or 73 or 74

76 13 and 75

77 exp primary health care/

78 ("primary health care" or "primary healthcare").tw.

79 primary care.tw.

80 general practice/

81 general practice*.tw.

82 family medicine.tw.

83 family medicine/

84 maternal child health care/

85 (mother and child health clinic*).tw.

86 (mother and child health center*).tw.

87 (mother and child health centre*).tw.

88 telemedicine/

89 telemedicine.tw.

90 telehealth/

91 telehealth.tw.

92 (ehealth or e-health).tw.

93 (mhealth or m-health).tw.

94 mobile health.tw.

95 smartphone/

96 smartphone.tw.

97 indigenous health care/

98 health services, indigenous/

99 aboriginal community controlled health*.tw.

100 (ACCHO or ACCHOs or ACCH or ACCHs).tw.

101 aboriginal medical service*.tw.

102 (AMS or AMSs).tw.

103 77 or 78 or 79 or 80 or 81 or 82 or 83 or 84 or 85 or 86 or 87 or 88 or 89 or 90 or 91 or 92 or 93 or 94 or 95 or 96 or 97 or 98 or 99 or 100 or 101 or 102

104 76 and 103

105 limit 104 to last 21 years

CINAHL Complete Search string

S1 TI infant* OR AB infant*

S2 (MH "Infant")

S3 TI child* OR AB child*

S4 TI preschool* OR AB preschool*

S5 TI pre-school* OR AB pre-school*

S6 TI nursery OR AB nursery

S7 TI p#ediatric OR AB p#ediatric

S8 TI “school student*” OR AB “school student*”

S9 TI “young person*” OR AB “young person*”

S10 TI "0 to 6 years" OR AB "0 to 6 years"

S11 TI "0-6 years" OR AB "0-6 years"

S12 S1 OR S2 OR S3 OR S4 OR S5 OR S6 OR S7 OR S8 OR S9 OR S10 OR S11

S13 TI “Middle ear disease*” OR AB “Middle ear disease*”

S14 TI “Middle ear inflammation” OR AB “Middle ear inflammation”

S15 TI “Middle ear infection” OR AB “Middle ear infection”

S16 TI “Middle ear effusion” OR AB “Middle ear effusion”

S17 TI “Glue ear” OR AB “Glue ear”

S18 (MH "Otitis Media+")

S19 TI “Otitis media*” OR AB “Otitis media*”

S20 TI ((OM OR OME)) OR AB ((OM OR OME))

S21 TI “Acute otitis media*” OR AB “Acute otitis media*”

S22 TI ((AOM OR AOMwoP OR AOMwiP)) OR AB ((AOM OR AOMwoP OR AOMwiP))

S23 TI “Chronic otitis media” OR AB “Chronic otitis media”

S24 TI “Chronic suppurative otitis media” OR AB “Chronic suppurative otitis media”

S25 TI CSOM OR AB CSOM

S26 TI “Persistent otitis media*” OR AB “Persistent otitis media*”

S27 TI POME OR AB POME

S28 TI “Serous otitis media” OR AB “Serous otitis media”

S29 TI “Secretory otitis media” OR AB “Secretory otitis media”

S30 (MH "Hearing")

S31 TI hearing OR AB hearing

S32 TI “Ear health” OR AB “Ear health”

S33 TI “Hearing difficult*” OR AB “Hearing difficult*”

S34 TI “ear discomfort” OR AB “ear discomfort”

S35 TI “Listening difficult*” OR AB “Listening difficult*”

S36 TI “Hearing loss” OR AB “Hearing loss”

S37 TI HL OR AB HL

S38 (MH "Hearing Loss, Conductive")

S39 TI "conductive hearing loss" OR AB “Conductive Hearing Loss”

S40 TI CHL OR AB CHL

S41 TI “Hearing impair*” OR AB “Hearing impair*”

S42 TI “Hearing problem*” OR AB “Hearing problem*”

S43 TI “hard of hearing” OR AB “hard of hearing”

S44 (MH "Otoscopy")

S45 TI “Otoscop*” OR AB “Otoscop*”

S46 TI “Pneumatic otoscop*” OR AB “Pneumatic otoscop*”

S47 TI “tympanometr*” OR AB “tympanometr*”

S48 TI “acoustic reflectometr*” OR AB “acoustic reflectometr*”

S49 (MH "Reflex, Acoustic")

S50 TI “acoustic reflex” OR AB “acoustic reflex”

S51 TI “Otoacoustic emission*” OR AB “Otoacoustic emission*”

S52 TI audiometry OR AB audiometry

S53 TI “Pure tone audiometry” OR AB “Pure tone audiometry”

S54 TI “Pure tone screen*” OR AB “Pure tone screen*”

S55 TI “Visual reinforcement Orientation Audiometry” OR AB “Visual reinforcement Orientation Audiometry”

S56 TI VROA OR AB VROA

S57 TI “Play audiometry” OR AB “Play audiometry”

S58 (MH "Tomography, Optical Coherence")

S59 TI “optical coherence tomography” OR AB “optical coherence tomography”

S60 TI OCT OR AB OCT

S61 TI “Hearing screen*” OR AB “Hearing screen*”

S62 TI “Hearing assess*” OR AB “Hearing assess*”

S63 (MH "Hearing Tests+")

S64 TI “hearing test*” OR AB “hearing test*”

S65 TI “hearing check*” OR AB “hearing check*”

S66 TI “ear check*” OR AB “ear check*”

S67 TI “ear health check*” AND AB “ear health check*”

S68 TI “hearing surveillance” OR AB “hearing surveillance”

S69 S13 OR S14 OR S15 OR S16 OR S17 OR S18 OR S19 OR S20 OR S21 OR S22 OR S23 OR S24 OR S25 OR S26 OR S27 OR S28 OR S29 OR S30 OR S31 OR S32 OR S33 OR S34 OR S35 OR S36 OR S37 OR S38 OR S39 OR S40 OR S41 OR S42 OR S43 OR S44 OR S45 OR S46 OR S47 OR S48 OR S49 OR S50 OR S51 OR S52 OR S53 OR S54 OR S55 OR S56 OR S57 OR S58 OR S59 OR S60 OR S61 OR S62 OR S63 OR S64 OR S65 OR S66 OR S67 OR S68

S70 S12 AND S69

S71 (MH "Primary Health Care")

S72 TI (("primary health care" OR "primary healthcare")) OR AB (("primary health care" OR "primary healthcare"))

S73 TI "primary care" OR AB "primary care"

S74 (MH "Family Practice")

S75 TI "general practice*" OR AB "general practice*"

S76 TI "family medicine" OR AB "family medicine"

S77 (MH "Maternal-Child Care")

S78 TI ("mother and child health clinic*") OR AB ("mother and child health clinic*")

S79 TI ("mother and child health center*") OR AB ("mother and child health center*")

S80 TI ("mother and child health centre*") OR AB ("mother and child health centre*")

S81 (MH "Telemedicine")

S82 TI telemedicine OR AB telemedicine

S83 (MH "Telehealth")

S84 TI telehealth OR AB telehealth

S85 TI ((ehealth OR e-health)) OR AB ((ehealth OR e-health))

S86 TI ((mhealth OR m- health)) OR AB ((mhealth OR m-health))

S87 TI "mobile health" OR AB "mobile health"

S88 (MH "Smartphone")

S89 TI smartphone OR AB smartphone

S90 (MH "Health Services, Indigenous")

S91 TI "aboriginal community controlled health*" OR AB "aboriginal community controlled health*"

S92 TI ((ACCHO OR ACCHOs OR ACCH OR ACCHs)) OR AB ((ACCHO OR ACCHOs OR ACCH OR ACCHs))

S93 TI "aboriginal medical service*" OR AB "aboriginal medical service*"

S94 TI ((AMS OR AMSs)) OR AB ((AMS OR AMSs))

S95 S71 OR S72 OR S73 OR S74 OR S75 OR S76 OR S77 OR S78 OR S79 OR S80 OR S81 OR S82 OR S83 OR S84 OR S85 OR S86 OR S87 OR S88 OR S89 OR S90 OR S91 OR S92 OR S93 OR S94

S96 S70 AND S95

S97 S70 AND S95 Limiters - Published Date: 20000101-

Database: EBM Reviews - Cochrane Central Register of Controlled Trials <June 2021>

Search Strategy:

----

1 infant*.tw.

2 child*.tw.

3 (preschool* or pre-school*).tw.

4 nursery.tw.

5 p?ediatric.tw.

6 school student*.tw.

7 young person*.tw.

8 "0 to 6 years".tw.

9 "0-6 years".tw.

10 1 or 2 or 3 or 4 or 5 or 6 or 7 or 8 or 9

11 Middle ear disease*.tw.

12 Middle ear inflammation.tw.

13 Middle ear infection.tw.

14 Middle ear effusion.tw.

15 Glue ear.tw.

16 Otitis media*.tw.

17 (OM or OME).tw.

18 Acute otitis media*.tw.

19 (AOM or AOMwoP or AOMwiP).tw.

20 Chronic otitis media.tw.

21 Chronic suppurative otitis media.tw.

22 CSOM.tw.

23 Persistent otitis media*.tw.

24 POME.tw.

25 Serous otitis media.tw.

26 Secretory otitis media.tw.

27 hearing.tw.

28 Ear health.tw.

29 Hearing difficult*.tw.

30 ear discomfort.tw.

31 Listening difficult*.tw.

32 Hearing loss.tw.

33 HL.tw.

34 Conductive Hearing Loss.tw.

35 CHL.tw.

36 Hearing impair*.tw.

37 Hearing problem*.tw.

38 hard of hearing.tw.

39 Otoscop*.tw.

40 Pneumatic otoscop*.tw.

41 tympanometr*.tw.

42 acoustic reflectometr*.tw.

43 acoustic reflex.tw.

44 Otoacoustic emission*.tw.

45 audiometry.tw.

46 Pure tone audiometry.tw.

47 Pure tone screen*.tw.

48 Visual reinforcement Orientation Audiometry.tw.

49 VROA.tw.

50 Play audiometry.tw.

51 optical coherence tomography.tw.

52 OCT.tw.

53 Hearing screen*.tw.

54 Hearing assess*.tw.

55 hearing test*.tw.

56 hearing check*.tw.

57 ear check*.tw.

58 ear health check*.tw.

59 hearing surveillance.tw.

60 11 or 12 or 13 or 14 or 15 or 16 or 17 or 18 or 19 or 20 or 21 or 22 or 23 or 24 or 25 or 26 or 27 or 28 or 29 or 30 or 31 or 32 or 33 or 34 or 35 or 36 or 37 or 38 or 39 or 40 or 41 or 42 or 43 or 44 or 45 or 46 or 47 or 48 or 49 or 50 or 51 or 52 or 53 or 54 or 55 or 56 or 57 or 58 or 59 (20238)

61 10 and 60

62 ("primary health care" or "primary healthcare").tw.

63 primary care.tw.

64 general practice*.tw.

65 family medicine.tw.

66 (mother and child health clinic*).tw.

67 (mother and child health center*).tw.

68 (mother and child health centre*).tw.

69 telemedicine.tw.

70 telehealth.tw.

71 (ehealth or e-health).tw.

72 (mhealth or m-health).tw.

73 mobile health.tw.

74 smartphone.tw.

75 aboriginal community controlled health*.tw.

76 (ACCHO or ACCHOs or ACCH or ACCHs).tw.

77 aboriginal medical service*.tw.

78 (AMS or AMSs).tw.

79 62 or 63 or 64 or 65 or 66 or 67 or 68 or 69 or 70 or 71 or 72 or 73 or 74 or 75 or 76 or 77 or 78

80 61 and 79

81 limit 80 to last 21 years

****

QUADAS-2 risk of bias ratings

QUADAS-2 risk of bias ratings for each included study

	Risk of bias					Applicability concerns		
**Key**<br>☺ Low risk<br>? Unclear risk<br>☹ High risk	Patient selection	Index test	Reference standard	Flow and timing		Patient selection	Index test	Reference standard
**Parent/caregiver concern**								
Lo et al, 2006	☺	☺	☺	?		☹	☺	☹
Engel et al, 2000	☺	☹	☹	☺		☺	☺	☺
Laine et al, 2010	☹	☹	☺	?		☹	☹	☺
Dickinson et al, 2018	☹	☹	☺	☹		☺	☺	☺
Cakabay et al, 2019	☹	?	☺	☺		☹	☹	☺
Swierniak et al, 2021	☺	☺	☺	?		☹	☺	☹
**Tools for screening ear health**								
Chianese et al, 2007	☺	☹	☹	☹		?	☺	☺
Helenius et al, 2012	☹	☺	☺	☺		☹	☺	☺
Abbott et al, 2014	☹	☹	☹	☹		☺	☹	☺
Puhakka et al, 2014	☹	☹	☺	?		☺	☺	☺
Alenezi et al, 2021	☹	☹	☹	☹		☹	☹	☹
Kleinman et al, 2021	☹	☹	☹	?		☹	☹	☹
**Tools for screening hearing**								
Newton et al, 2001	☹	☹	☺	?		☹	☺	☺
Mahomed-Asmail et al, 2016	?	☺	?	?		☺	☺	☺
Ramkumar et al, 2018	?	☺	☺	?		☺	☺	☺
Mealings et al, 2020	☹	☺	☺	?		☺	☺	☺
Orzan et al, 2021	☹	☹	☺	☹		☹	☺	☺

Agree II Guidelines ratings

AGREE II Guidelines ratings	Domain 1 Scope and purpose			Domain 2 Stakeholder involvement			Domain 3 Rigour of development			Domain 4 Clarity of presentation			Domain 5 Applicability			Domain 6 Editorial independence		
**Guideline**	**Sum**	**Mean**	**Score**	**Sum**	**Mean**	**Score**	**Sum**	**Mean**	**Score**	**Sum**	**Mean**	**Score**	**Sum**	**Mean**	**Score**	**Sum**	**Mean**	**Score**
American Academy of Audiology Clinical Practice Guidelines Childhood Hearing Screening (2011)	36	6.0	**83%**	22	3.7	**44%**	48	3.0	**33%**	41	6.8	**97%**	45	5.6	**77%**	4	1.0	**0%**
NACCHO and RACGP National guide to a preventive health assessment for Aboriginal and Torres Strait Islander people (2018)	42	7.0	**100%**	26	4.3	**56%**	71	4.4	**57%**	40	6.7	**94%**	36	4.5	**58%**	28	7.0	**100%**
Menzies School of Health Research. Otitis Media Guidelines for Aboriginal and Torres Strait Islander Children (2020)	40	6.7	**94%**	22	3.7	**44%**	86	5.4	**73%**	38	6.3	**89%**	33	4.1	**52%**	11	2.8	**29%**
Danish guidelines on management of otitis media in preschool children (2016)	42	7.0	**100%**	27	4.5	**58%**	110	6.9	**98%**	41	6.8	**97%**	9	1.1	**2%**	20	5.0	**67%**
American Academy of Pediatrics Year 2007 position statement: Principles and guidelines for early hearing detection and intervention programs	28	4.7	**61%**	18	3.0	**33%**	66	4.1	**52%**	26	4.3	**56%**	31	3.9	**48%**	10	2.5	**25%**
Clinical Practice Guideline: Otitis Media with Effusion (Update). Rosenfeld et al. (2016)	40	6.7	**94%**	28	4.7	**61%**	97	6.1	**84%**	37	6.2	**86%**	32	4.0	**50%**	24	6.0	**83%**
American Academies of Family Physicians, Otolaryngology-Head and Neck Surgery, and Pediatrics. Otitis Media with Effusion Clinical Practice Guideline (2004)	42	7.0	**100%**	29	4.8	**64%**	96	6.0	**83%**	30	5.0	**67%**	26	3.3	**38%**	4	1.0	**0%**
Korean clinical practice guidelines: otitis media in children (2012)	39	6.5	**92%**	24	4.0	**50%**	84	5.3	**71%**	31	5.2	**69%**	25	3.1	**35%**	4	1.0	**0%**

GRADE ratings by Ear Health and Hearing Check domain

Domain	Outcome	Papers	Risk of Bias	Inconsistency	Indirectness	Imprecision	Publication Bias	Overall Certainty
**Hearing**	Parent concern re hearing	Swierniak et al 2021<br>Lo et al 2006<br>Dickinson et al 2018	No	Very Serious	Serious	Cannot be rated	Undetected	Low
**Ear Health**	Parent reported symptoms of OME	Laine 2010<br>Cakabay et al	Serious	Cannot be rated	Serious	No	Strongly Suspected	Very low
	Video Otoscopy	Alenezi et al 2021	Very Serious	Cannot be rated	Serious	Cannot be rated	Strongly Suspected	Very low
	Smartphone Otoscopy	Kleinman et al. 2021.	Serious	Cannot be rated	Serious	Serious	Strongly Suspected	Very low
	Tympanometry	Puhakka et al. 2014<br>Helenius et al. 2012<br>Chianese et al. 2007<br>Abbott et al	Very Serious	Cannot be rated	Serious	No	Strongly Suspected	Very low
	SGAR	Puhakka et al. 2014<br>Chianese et al. 2007	Serious	Cannot be rated	No	No	Undetected	Low
**Hearing Sensitivity**	OAE	Ramkumar et al 2018	Serious	Cannot be rated	No	No	Strongly Suspected	Low
	Automated Tests	Mealings et al. 2020<br>Mahomed-Asmail et al. 2016	Serious	Very Serious	No	Cannot be rated	Strongly Suspected	Very low
	Whispered Voice Test	Pirozzo et al. 2003	Very Serious	Serious	Very Serious	Serious	Undetected	Very low
**Listening Skills Development**	Listening skills development monitoring	Orzan et al<br>Newton et al 2001	Very Serious	Serious	Serious	Cannot be rated	Strongly Suspected	Very low
